# Alternative splicing of *jnk1a* in zebrafish determines first heart field ventricular cardiomyocyte numbers through modulation of hand2 expression

**DOI:** 10.1371/journal.pgen.1008782

**Published:** 2020-05-18

**Authors:** Adrian Santos-Ledo, Sam Washer, Tamil Dhanaseelan, Lorraine Eley, Ahlam Alqatani, Paul W. Chrystal, Tania Papoutsi, Deborah J. Henderson, Bill Chaudhry

**Affiliations:** Biosciences Institute, Faculty of Medicine, International Centre for Life, Newcastle University, United Kingdom; Indiana University Purdue University at Indianapolis, UNITED STATES

## Abstract

The planar cell polarity pathway is required for heart development and whilst the functions of most pathway members are known, the roles of the *jnk* genes in cardiac morphogenesis remain unknown as mouse mutants exhibit functional redundancy, with early embryonic lethality of compound mutants. In this study zebrafish were used to overcome early embryonic lethality in mouse models and establish the requirement for Jnk in heart development. Whole mount in-situ hybridisation and RT-PCR demonstrated that evolutionarily conserved alternative spliced *jnk1a* and *jnk1b* transcripts were expressed in the early developing heart. Maternal zygotic null mutant zebrafish lines for *jnk1a* and *jnk1b*, generated using CRISPR-Cas9, revealed a requirement for *jnk1a* in formation of the proximal, first heart field (FHF)-derived portion of the cardiac ventricular chamber. Rescue of the *jnk1a* mutant cardiac phenotype was only possible by injection of the *jnk1a EX7 Lg* alternatively spliced transcript. Analysis of mutants indicated that there was a reduction in the size of the *hand2* expression field in *jnk1a* mutants which led to a specific reduction in FHF ventricular cardiomyocytes within the anterior lateral plate mesoderm. Moreover, the *jnk1a* mutant ventricular defect could be rescued by injection of *hand2* mRNA. This study reveals a novel and critical requirement for *Jnk1* in heart development and highlights the importance of alternative splicing in vertebrate cardiac morphogenesis. Genetic pathways functioning through *jnk1* may be important in human heart malformations with left ventricular hypoplasia.

## Author summary

The planar cell polarity pathway is a genetic cascade important for normal development of the heart. The *jnk* gene family is known to act within this pathway but its specific role in heart development has been difficult to establish using mouse mutants because genes within the family overlap in function and embryos mutant for more than one *jnk* gene die before the heart is fully formed. In these studies, we used zebrafish to determine the role of *jnk1* in heart development and found that a specific gene transcript from zebrafish *jnk1a* is needed to produce the full complement of cardiomyocytes that form the first part of the ventricular heart chamber. We show that this happens because *jnk1a* is required for the normal expression of *hand2*, a gene required to make ventricular heart cells. Furthermore, we can fully reverse the abnormalities in *jnk1a* embryos by increasing *hand2* gene activity. This part of the zebrafish ventricular chamber is equivalent to the left ventricle in humans and understanding how *jnk* genes work may help understand why some human heart malformations are associated with an underdeveloped left ventricle.

## Introduction

Over 1% of the population have structural congenital heart disease (CHD) [1]. Several studies have suggested genes associated with syndromic heart disease [2–5], but for the majority of patients with isolated CHD, causative genes remain elusive. An alternative approach to population-based genomic studies is to understand how the heart forms and to identify pathways and genes that are relevant to CHD. One genetic pathway that has been shown to be important in pre-clinical models is the non-canonical Wnt, planar cell polarity (PCP) pathway. Disruption of key genes such as *Vangl2*, *Dvl2* and *Celsr1* have been shown to cause cardiac malformations (reviewed in [6]), specifically involving addition of second heart field precursors (SHF) [7]. Inactivation of downstream pathway effectors, such as Rac1 [8,9] and Rho Kinase (ROCK) 1 and 2 [10] also produce cardiac defects.

The c-Jun N-terminal kinases (JNK1, 2 and 3) are recognised as mediators of PCP signalling, acting downstream of RhoA, but are also members of the mitogen activated protein kinase (MAPK) family. These stress-responsive serine/threonine kinases are implicated in a diverse range of biological processes including embryonic development, adult onset brain disease, tumour survival and metastatic spread via control of cell proliferation, cell death, movement, and direct regulation of gene expression (reviewed in [11]). Jnk is phosphorylated by MAP kinase kinases (MAP2K), which in turn are activated by MAP kinase kinase kinases (MAP3K) [12]. Jnk also integrates signals from the RAS and AKT pathways. For example, the R497Q mutation in *SOS1* found in patients with Noonan syndrome, has been shown to directly activate Jnk [13]. Up to 95% of all human genes, including *Jnk*, undergo evolutionarily conserved alternative splicing [14], increasing the diversity of gene products. In *Jnk*, alternative usage of two neighbouring central exons in association with truncated or extended C-termini, creates four alternative peptide structures. An alternative translation initiation site for *Jnk3* increases the range of transcripts for that gene [15,16]. *Jnk1* and *Jnk2* are co-expressed in almost all cells and tissues, whilst *Jnk3* is reported to be expressed predominantly in the brain, testes and heart [17]. This redundancy of expression, and probably function, is one reason why identifying the roles of *JNK* has been difficult, particularly those in cardiovascular development. Differential regulation of gene expression by different splice isoforms of *Jnk1* has been studied in NIH3T3 fibroblasts [18], but tissue specific expression of the alternatively spliced transcripts has not been examined in animal studies and little is known of their roles in heart development [11]. The *Jnk1* null mouse is viable and fertile, with no obvious malformations, but has abnormal differentiation of T-helper cells and reduced adipose tissue [17]. Similarly, the *Jnk2* null mouse also has abnormalities of T-cell function and abnormal brain development [17,19,20]. In contrast, the *Jnk3* null mouse is essentially normal and despite the specific expression of *Jnk3* within the central nervous system, has normal brain histology [17,19]. Attempts to unravel these roles in compound null mutants have met limited success. Mice null for both *Jnk1* and *Jnk2* die by embryonic day E11, with evidence of extensive apoptosis in the brain. No specific cardiac abnormalities were noted although some embryos had cardiac dilatation, interpreted to be a non-specific finding [19].

The zebrafish has become established as an important pre-clinical model, especially in developmental studies, because of the high degree of conservation of vertebrate genes and developmental processes. The unique combination of transgenic reporter lines, transparent embryos, and ease of genetic manipulation through morpholino and CRISPR-Cas9 genome editing give practical advantages over other laboratory animals [21]. Zebrafish are particularly useful for cardiovascular developmental studies as the embryos can survive without a functional circulation for up to one week [22]. Although the zebrafish has only a single atrium and ventricle, the initial fundamental processes of vertebrate heart formation are conserved. An initial heart tube forms from migrating first heart field (FHF) progenitors and further addition of SHF progenitors augments the ventricular mass and atrium [21]. Thus, although not septated, the zebrafish ventricle contains a proximal FHF-derived portion that is equivalent to the FHF-derived left ventricle in mammals, and a distal SHF-derived component which is the equivalent to the right ventricle [23,24]. The transcriptional regulation of FHF cardiomyocyte specification within the anterior lateral plate mesoderm (ALPM) is well understood and requires overlapping expression of key transcriptional regulators including *nkx2*.*5*, *gata4* and also *hand2*, which plays a permissive role in cardiomyocyte formation [25,26]. A complicating factor in zebrafish genetics is a genome duplication event [27], such that some genes persist as duplicated paralogs, often with sub-functionalisation and different expression patterns. For example, Ensembl suggests that there is only one persisting functional gene for *jnk2* and *jnk3*, but *jnk1* remains duplicated as *jnk1a* and *jnk1b*.

In this study we demonstrate a requirement for the *jnk1* gene in vertebrate heart development utilising the specific advantages of zebrafish as a model organism and overcoming the early embryonic death that has limited mouse studies. We show that the landscape of alternatively spliced *jnk1a* and *jnk1b* transcripts during embryogenesis is dynamic and that different changes are seen within the developing heart. Using CRISPR-cas9-derived null mutants and mRNA rescue studies, we show a specific requirement for the *jnk1a Ex7 Lg* transcript in development of the FHF-derived ventricle; the evolutionary equivalent of the left ventricle in man. We go on to determine that the FHF-ventricular hypoplasia found in mutants originates from a reduction in the number of specified cardiomyocytes in the ALPM, and this is due to a reduction in the expression field of the key transcriptional regulator *hand2*. Finally, we confirm these findings by rescuing the cardiomyocyte deficiency in *jnk1a* mutant embryos with *hand2* mRNA.

## Results

### Human alternative splicing patterns are conserved for the duplicated zebrafish *jnk1a/jnk1b* genes

**Fig 1:**
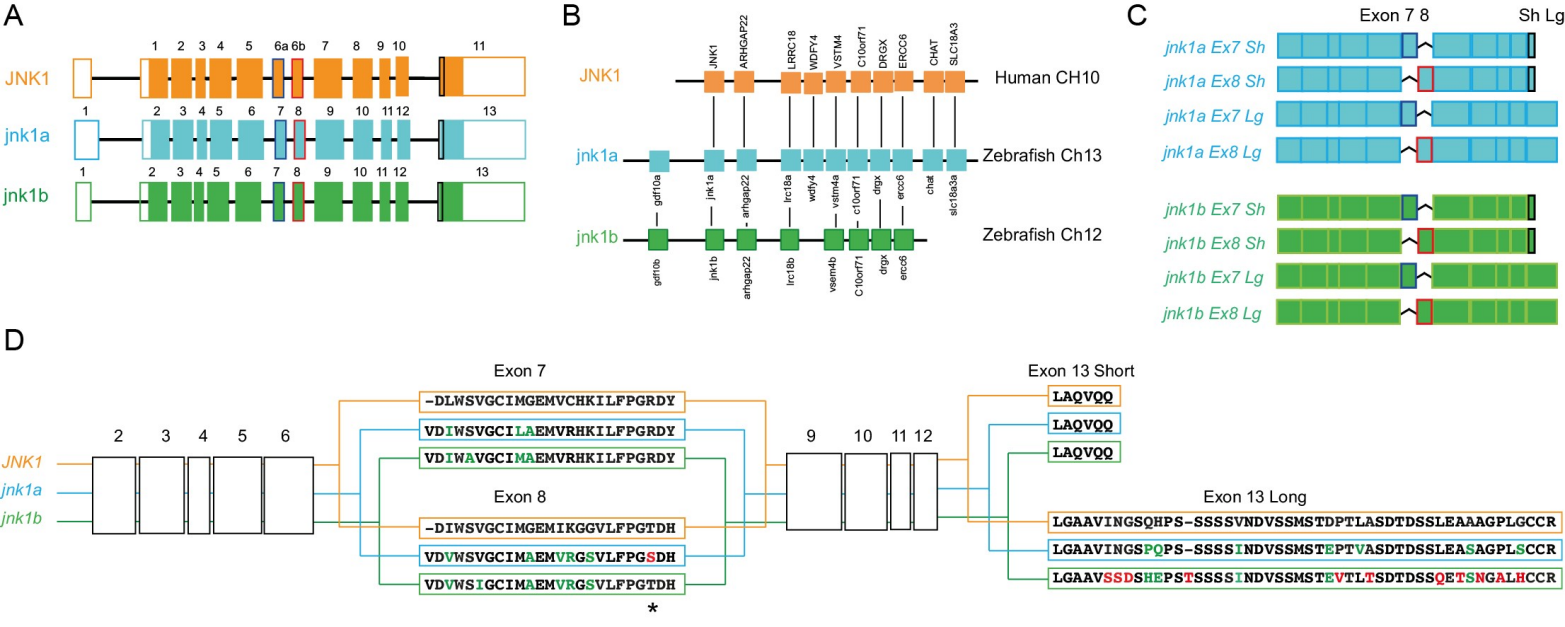
Zebrafish jnk1a and jnk1b are paralogs of human JNK1. (**A**) Despite different exon numbering i.e. 6a/6b in human and 7/8 in zebrafish orthologues, exon structure is entirely conserved. (**B**) *jnk1a*, *jnk1b* and *JNK1* lie in syntenic chromosomal regions. (**C**) Cartoon showing how the 8 *jnk1a/b* transcripts result from alternate exon 7/8 usage and differing C-terminal extension. (**D**) Translation of zebrafish *jnk1a* and *jnk1b* transcripts showing amino acids from alternatively spliced exons. Black text indicates identical amino acids, green text indicates favourable amino acid substitutions and red text divergent amino acid residues. Both *jnk1a* and *jnk1b* genes are capable of producing Ex7 and short C-terminus containing transcripts that fully match human *JNK1* transcripts. However, whilst *Ex8* derived from *jnk1b* matches the human peptide, the *Ex8* from *jnk1a* is divergent and contains a serine rather than threonine residue (*). In contrast whilst the long C-terminal extension provided by *jnk1a* matches the human, the *jnk1b* long terminal extension is highly divergent and differs by 9/39 amino acids including insertion of an additional threonine residue. See S1 Fig for full peptide sequences.

In previous zebrafish studies neither the duplication of *jnk1* nor the existence of alternatively spliced transcripts were evaluated [28]. Our bioinformatic analysis of the zebrafish genome (Ensembl assembly GRCz11 [29]) indicated that the *jnk1a* and *jnk1b* genes are duplicated paralogues of human *JNK1*, possessing conserved exon structure (Fig 1A) and residing on syntenic chromosomal regions (Fig 1B). Full-length transcripts, sub-cloned from pooled 24, 48 and 72 hpf zebrafish embryos, revealed full conservation of the alternative splicing patterns seen in human *JNK1* (Fig 1C). Both *jnk1a* and *jnk1b* provided four transcript variants through alternative exon 7/8 usage and an alternative splice re-entry site encoding short (Sh) and long (Lg) C-terminal peptides (Fig 1C and 1D and S1 Fig). These exons and C-terminal extensions directly correspond to exon 6a/6b and the p46 or p54 C-terminal extension variants of human *JNK1* (Fig 1C and 1D and S1 Fig) [11]. Comparison of the predicted peptide sequences of the zebrafish jnk1 proteins with human JNK1 [30] indicated a high degree of overall structural conservation, but potential sub-functionalisation in the zebrafish through variations in amino acid residues within the alternatively spliced exons (Fig 1D and S1 Fig). The zebrafish *jnk1a* gene was capable of producing both Ex7 Lg and Sh products equivalent to human *JNK1 Ex6a p46* and *JNK1 Ex6a p54* C-terminal extension variants, but not Ex8 products equivalent to human *JNK1 Ex6b p46* and *p54* C-terminal extension variants as *jnk1a* encoded a serine instead of threonine residue within exon 8. In *jnk1b*, the short C terminal extension is highly conserved. However, the *jnk1b* long C-terminal extension is extremely divergent from the human p54 equivalents, suggesting a species-specific role or simply degenerate sequence (Fig 1D and S1 Fig). Thus, jnk1b Ex7Sh and jnk1b Ex8Sh peptides are extremely similar to human JNK1 Ex6a p46 and JNK1 Ex6b p54 peptides, but the jnk1b Lg peptides are not. To further understand how transcript expression is controlled by *jnk1a* and *jnk1b* gene duplication we needed to quantify all eight alternatively-spliced zebrafish transcripts.

### Quantification of *jnk1a*/*jnk1b* transcripts and splicing

**Fig 2:**
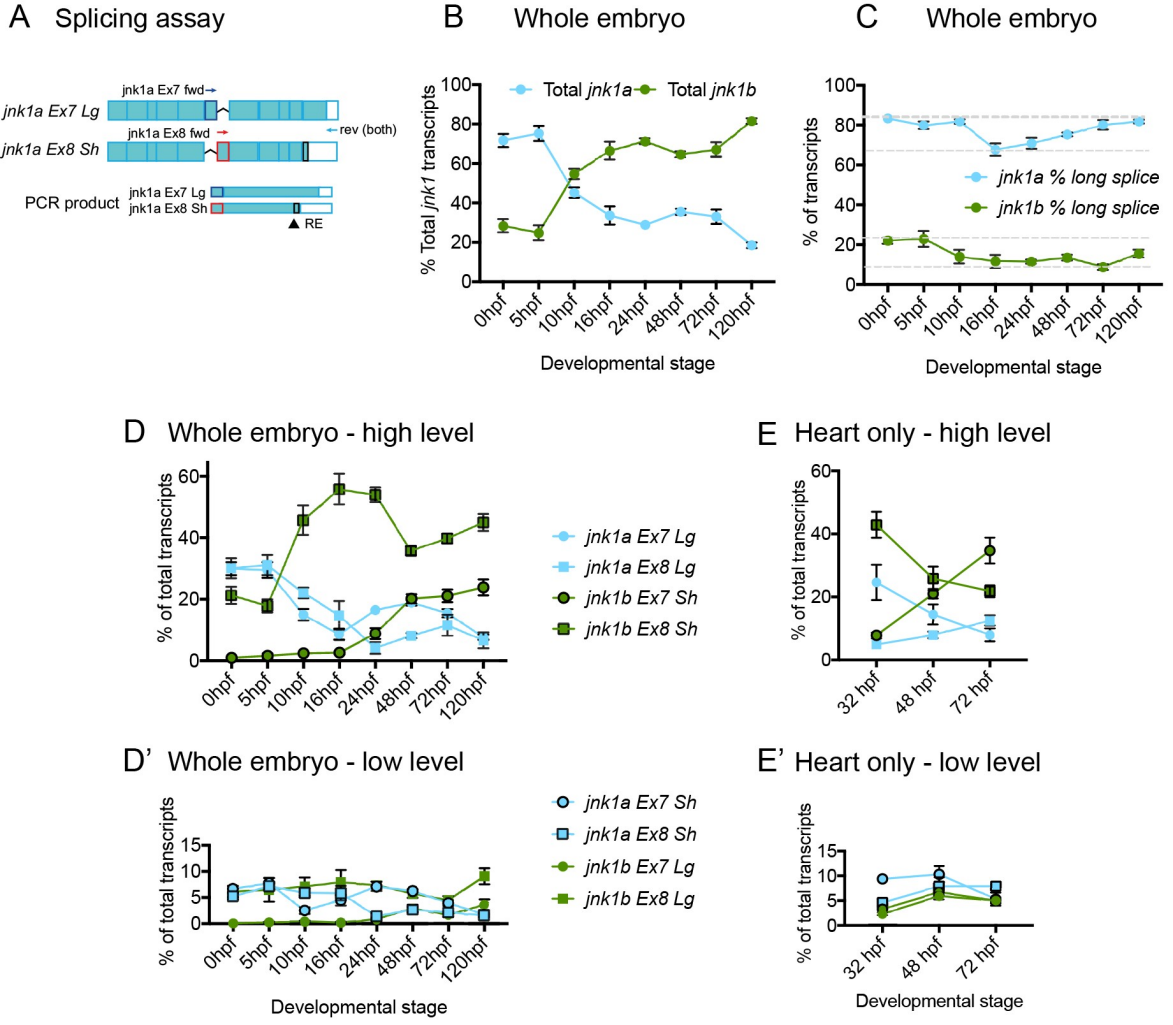
Semi-quantitative *jnk1a/b* transcript assay. (**A**) Schematic of assay for *jnk1a* (see methods for full details). RNA is extracted and a cDNA pool produced. PCR within linear phase performed using forward (fwd) primers specific for *jnk1a*/*1b* exons 7/8 and common reverse (rev) primers. The fraction with C-terminal extension is determined by restriction enzyme (RE) digestion. (**B**) Percentage of total *jnk1a* and *jnk1b* transcripts during embryonic development. 14 ss = 16 hpf. (**C**) Percentage of total Lg *jnk1a* and *jnk1b* transcripts during embryonic development. (**D and D'**) Proportion of each individual alternatively spliced transcript during development in whole embryo. (**E and E'**) Proportion of each individual transcript within isolated whole heart during development. *jnk1a* = blue, *jnk1b* = green, *Ex7* = circle, *Ex8* = square, *Sh* = outline black and *Lg* = no outline. n = 6–8 at all time points.

To measure the relative expression of the alternatively spliced transcripts and evaluate changes in splicing we designed a semi-quantitative RT-PCR/restriction enzyme assay (Fig 2A and S2 Fig for validation of assay) [31]. Analysis of the proportions of *jnk1a*/*jnk1b* transcripts in whole embryos revealed changing differential gene expression between *jnk1a* and *jnk1b* but also changes in alternative splicing. Prior to gastrulation, 80% of *jnk1* zebrafish transcripts isolated from whole embryos originated from *jnk1a*. However, after gastrulation *jnk1b* transcripts became increasingly abundant and by 120hpf, 80% of transcripts originated from *jnk1b* (Fig 2B). The majority of the long C-terminal extension transcripts originated from *jnk1a* and most of the short C-terminal extension transcripts from *jnk1b* (Fig 2C). Thus, changes in the proportions of long or short C terminal extension were regulated predominantly by changes in gene expression rather than alternative splicing. However, alternative splicing did dictate the proportions of different transcripts containing either Ex7 or Ex8 in the *jnk1* genes. Changes in splicing were less marked for *jnk1a* transcripts, which had approximately equal representation of exons 7 and 8. In contrast, the majority of *jnk1b* transcripts contained *Ex8* during the first 48hrs of development but after this the levels of the *jnk1b Ex7* transcript greatly increased (Fig 2D). All other transcripts persisted at low level (Fig 2D'). Since these whole embryo data are dominated by expression in the forming brain and tail muscles, we performed the splicing assay on isolated whole hearts [32] to better understand expression during cardiac development. Key stages of morphogenesis were evaluated, at 32hpf, shortly after the linear heart tube has formed from FHF precursors and then at 48hpf and 72hpf, when addition of the SHF is complete [23,33] (Fig 2E and 2E'). At 32hpf, as in the whole embryo, *jnk1b Ex8Sh* was the most abundant transcript (Fig 2D and 2E). By 72hpf days a moderate increase in proportion of *jnk1b Ex7Sh* in the whole embryo indicated changes in alternative splicing (Fig 2D). But it was only within the heart that this resulted in *jnk1b Ex7Sh* becoming the most abundant transcript (Fig 2E). During this period the proportion of *jnk1a Ex7Lg* and *jnk1a Ex8Lg* transcripts fell within the whole embryo. Importantly, the proportions of *jnk1a* transcripts containing Ex7 versus Ex8 remained relatively unchanged in the whole embryo and there was no increase in *Ex7* transcripts within the heart, indicating a novel alternative splicing event was limited to *jnk1b* (Fig 2D and 2E). Thus, in zebrafish expression of long terminal extension transcripts is dictated by relative expression levels of *jnk1a* and *jnk1b genes*. Alternative splicing, whilst occurring to a minor extent in *jnk1a*, is a significant feature of *jnk1b* and controls the switch from *jnk1b Ex8* to *jnk1b Ex7* expression in the heart and greater expression in the embryo as a whole.

### Expression of *jnk1a* and *jnk1b* transcripts and the developing heart

**Fig 3:**
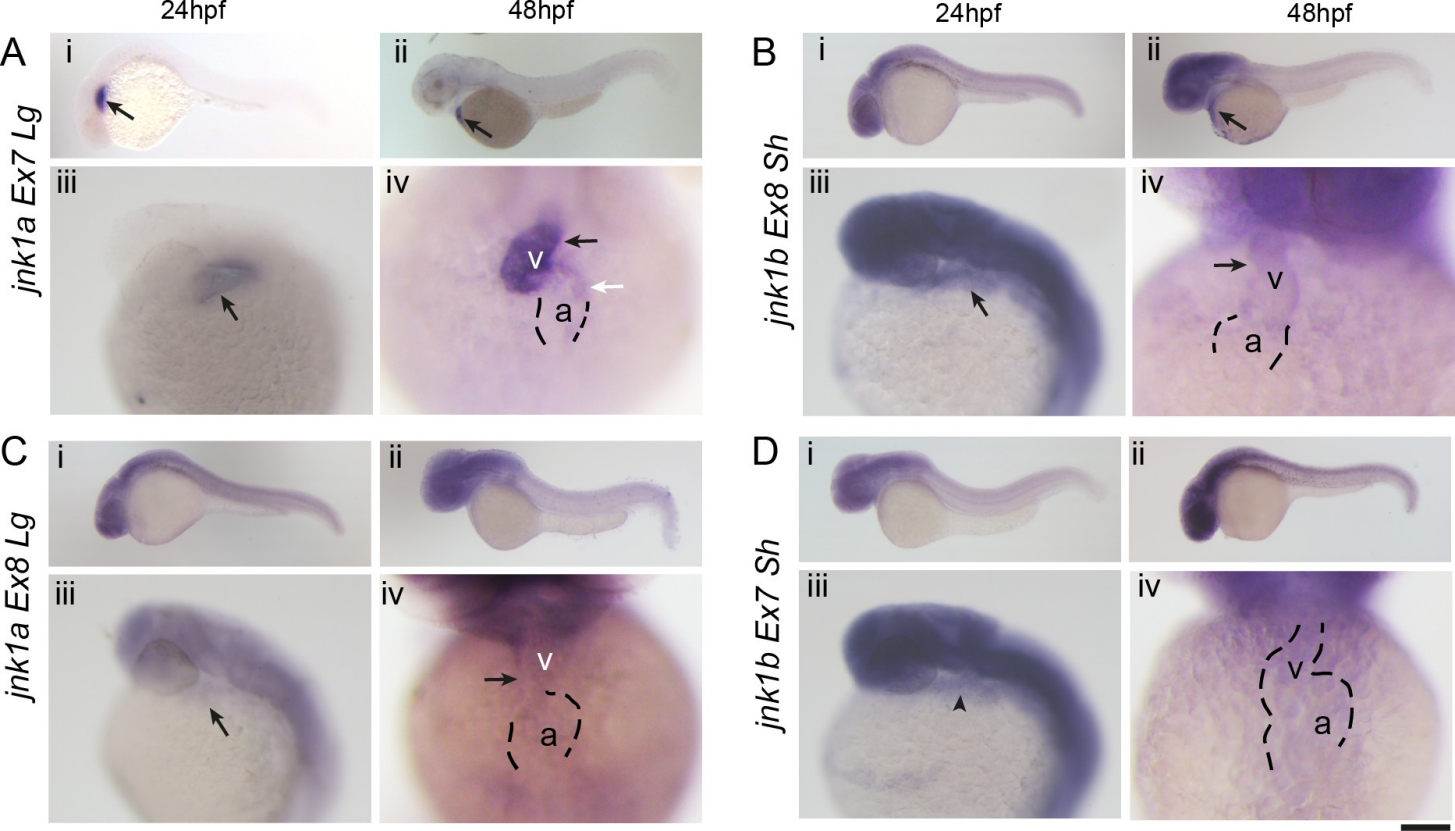
Expression of *jnk1a/b* transcripts in development. (**A-D**) Expression patterns of the four *jnk1* transcripts most highly expressed in the heart (arrows) at 24hfp (i, iii), 48hpf (ii, iv) from lateral (i, ii) and left oblique views (iii, iv). (**A**) *jnk1a Ex7Lg* is expressed in the cardiac cone (arrow) at 24hpf. At 48hpf strong expression is seen in the proximal part of the cardiac ventricle (v; black arrow) and weak expression is seen in some cells in the outer curvature of the atrium (a; white arrow). (**B**) At 24hpf *jnk1b Ex8Sh* expression in the cardiac cone is difficult to determine due to expression in overlapping head structures (i, iii), but is clearly seen in the outflow tract (arrow), and lower level in the ventricle, by 48hpf (ii-iv). (**C**) *jnk1a Ex8Lg* is expressed at low level in both the atrium and ventricle at 24 and 48 hpf. (**D**) *jnk1b Ex7Sh* is expressed in the region of the heart but is also expressed in surrounding tissues. Scale bar in panels i and ii 1mm, panels iii, iv 0.5mm.

To compliment these quantitative analyses spatial analysis by WISH was carried out using full-length riboprobes and high-stringency conditions (Fig 3 and S3 Fig). Prior to (6hpf) and immediately after (10hpf) gastrulation all the transcripts appear to be widely expressed throughout the embryo and thereafter expressed in the brain and somites (S3 Fig). However, closer examination of the expression patterns of the most abundant transcripts identified from the splicing assay, revealed a highly specific expression for *jnk1a Ex7Lg* only within the heart at 24hpf (Fig 3A i,iii). By 48hpf the *jnk1a Ex7Lg* transcript was clearly localised to the proximal part of the developing ventricular chamber, with minimal, localised expression in the adjacent part of the developing atrium (Fig 3A ii,iv).

The most abundant transcript within the heart, *jnk1b Ex8Sh*, was also clearly seen within the heart at 48hpf (Fig 3B ii,iv), but was less clear at 24hpf because of strong expression in overlying head tissues (Fig 3B i,iii). The weak expression patterns of *Jnk1a ex8Lg* (Fig 3C) and *Jnk1b ex7Sh* (Fig 3D) within the heart at 24 and 48hpf were in keeping with lower levels of expression as indicated by the RT-PCR splicing assay. Examination of *jnk2* and *jnk3* expression by RT-PCR and WISH to detect all transcripts indicated that these gene products were not expressed in the developing heart before 48hpf (S3D Fig)

Although there is clearly the risk of cross reactivity for these riboprobes, taken together these experiments, especially the highly specific *jnk1a Ex7Lg* expression pattern, suggest that *jnk1a and jnk1b* genes may play roles in early heart development through expression of specific alternatively spliced transcripts.

### *jnk1a* and *jnk1b* mutants are viable and fertile

**Fig 4:**
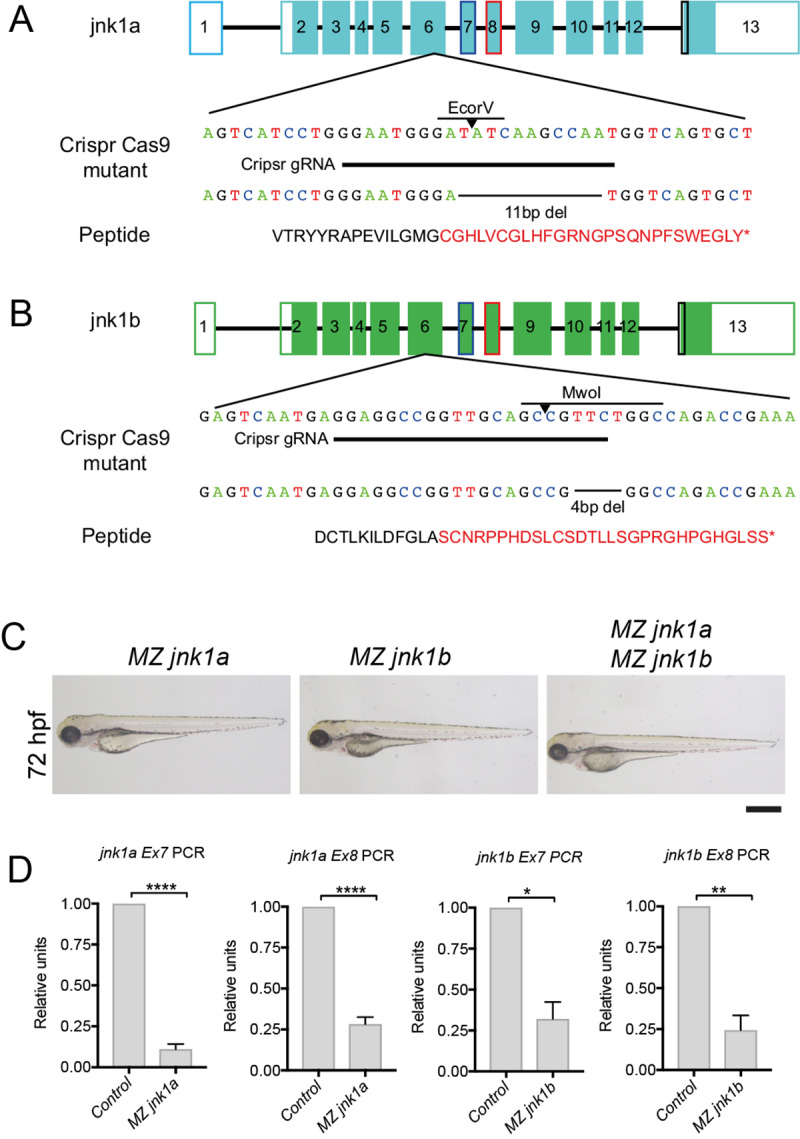
*jnk1a/b* mutants. Sequences of genomic DNA for *jnk1a* **(A)** and *jnk1b* **(B)** showing targeting sequence for CRISPR-Cas9 guide RNA; deletions produced; translated peptide sequence (* = stop); and restriction enzymes that identify mutants. Both *jnk1a* and *jnk1b* mutations disrupt sequence within exon 6 and predicted to lead to loss of all alternatively spliced transcripts by nonsense-mediated decay. (**C**) *Maternal zygotic (MZ) jnk1a*, *MZjnk1b* and *MZjnk1a/ MZjnk1b* mutants all appear grossly normal and are fertile. **(D**) RT-PCR using primers specific for *jnk1a* and *jnk1b* exon 7 and 8 show nonsense-mediated decay in *MZjnk1a* and *MZjnk1b mutants*. * = p<0.05, ** = p<0.01, **** = p<0.0001. n = 3 (3 separate clutches of 40 embryos obtained from different pairs of fish). Scale bar 1mm.

To understand the roles of *jnk1a and jnk1b* in heart development we produced null mutants using CRISPR-Cas9. Missense mutations creating premature stop codons were confirmed by sequencing genomic DNA (Fig 4A and 4B). After initial outcrossing, heterozygote mutants were in-crossed and resulted in zygotic (Z) null mutants that were fertile and of normal appearance. Further in-crosses produced homozygous maternal zygotic (MZ) null lines, whose eggs contained no maternally deposited *jnk* mRNA. These *MZjnk1a*, *MZjnk1b* and *MZjnk1a*/*MZjnk1b* zebrafish appeared grossly normal with normal behaviour and fertility (Fig 4C). Nonsense-mediated decay was confirmed by RT-PCR in MZ null mutants (Fig 4D). Importantly evaluation of *jnk1a Ex7*, *jnk1a Ex8*, *jnk1b Ex7* and *jnk1b Ex8* transcript levels in *MZjnk1a*, *MZjnk1b* and *MZjnk1a/MZjnk1b* embryos excluded any genetic compensation in the CRISPR mutants from *jnk1a* and *jnk1b* respectively (S4 Fig)

### Loss of *jnk1a* leads to ventricular hypoplasia

**Fig 5:**
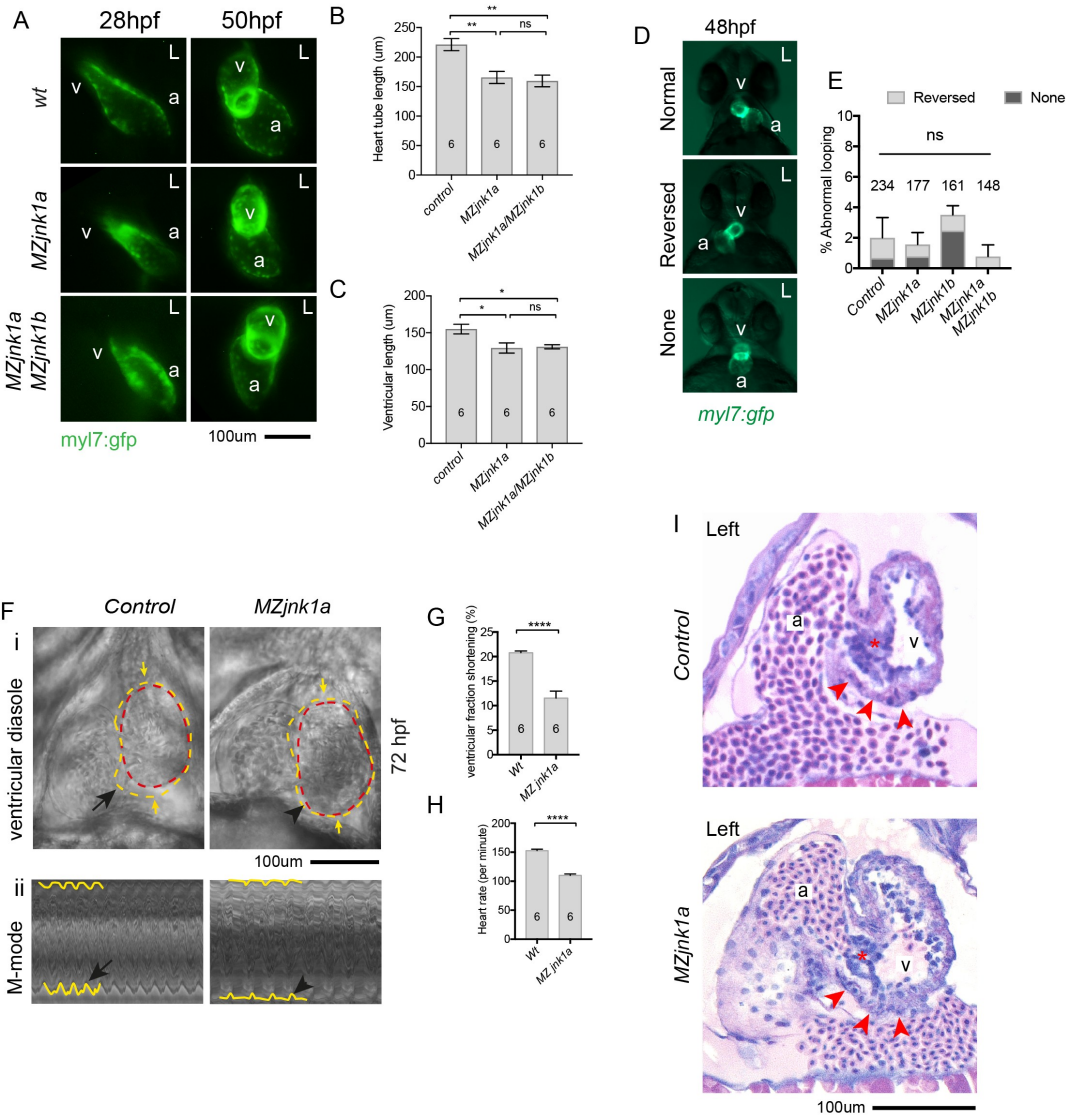
Cardiac abnormalities in *MZjnk1a* mutants. **(A)** Live imaging of wildtype, *MZjnk1a* and *MZjnk1a/ MZjnk1b* mutants carrying *myl7*:*gfp* transgene at 28hpf and 50hpf. (**B**) overall primary heart tube length is reduced in *MZjnk1b* and *MZjnk1a/ MZjnk1b* embryos at 28hpf and (**C**) ventricular length is reduced at 50hpf. (**D**) Appearances of normal, reversed and no cardiac looping visualised by the *myl7*:*gfp* transgene in wildtype embryos. (**E**) Minimal levels of disordered cardiac looping in *MZjnk1a* and *MZjnk1b* mutants, alone and in combination. (**F**) Cardiac contractile function in *MZjnk1a* mutants and controls at 72 hpf. See S1 Movie, control, and S2 Movie, *MZjnk1a* mutant. External margin of the ventricle identified in diastole (yellow dashed line) and systole (red dashed line). i) Ventricular contraction is impaired in the FHF-derived ventricular segment (black arrowhead). ii) "M-mode" representations of the movies are obtained by resampling between the yellow arrows. Hypokinesis in the *MZjnk1a* heart (black arrowhead) can be seen, in comparison to the normal waveform of contraction in the control heart (black arrow). (**G**) Reduced fractional shortening and (**H**) heart rate in *MZjnk1a* mutants at 72 hpf. (**I**) Histological sections through heart of wildtype and *MZjnk1a* embryos in plane of imaging in (F). Red arrowheads indicate hypokinetic FHF ventricular segment, red asterix indicates atrioventricular valve. a = atrium, v = ventricle, L = left. * = p<0.05, ** = p<0.01, **** = p<0.0001, ns = not significant.

Despite the lack of any gross somatic phenotype in these mutants, the highly specific expression pattern of *jnk1a Ex7Lg* (Fig 3A) led us to examine heart development in more detail. Formation of the initial heart tube by migrating FHF cardiomyocytes is complete by 28hpf and imaging of live embryos expressing GFP within cardiomyocytes suggested that the heart tube was reduced in length in *MZjnk1a* mutants at that time (Fig 5A). Simple measurement of the length of the heart tube confirmed this (Fig 5B). The heart appeared of normal size in *MZjnk1b* mutants and there was no further size reduction in *MZjnk1a*/*MZjnk1b* double mutants, above that seen in the *MZjnk1a* mutants. Imaging at 50hpf revealed a reduction in the linear length of the ventricular chamber (Fig 5A–5C). Wholemount immunofluorescent labelling with antibodies to identify ventricular and atrial chambers also confirmed the reduction in ventricular length (S5 Fig).

As the chambers form, looping of the heart tube takes places to position the atrium to the left side of the more centrally placed ventricle. In 1–2% of normal embryos looping is reversed or both the chambers remain in the midline. Examination of *MZjnk1a*, *MZjnk1b* and *MZjnk1a*/*MZjnk1b* mutants demonstrated no abnormalities of looping (Fig 5D and 5E).

We examined heart function at 72hpf in control and *MZjnk1a* mutant embryos to see if there might be persisting functional consequences from this reduction in ventricular chamber size. Using video microscopy, it was evident that the proximal ventricular component, adjacent to the atrioventricular valve, was of reduced size and hypokinetic in the *MZjnk1a* mutant compared with their control counterparts (Fig 5F) leading to a reduction in ventricular fractional shortening (Fig 5G). There was also a significant reduction in heart rate in the mutant embryos at the same stage (Fig 5H). Histological resin sections taken through *MZjnk1a* hearts at 72hpf revealed no differences in the ventricular myocardial wall in the hypokinetic ventricular segment and the atrioventricular valve appeared of normal morphology (Fig 5I). Thus, *jnk1a* is required for normal ventricular development and in *MZjnk1a* mutants the FHF-derived proximal ventricular component has persisting dyskinesis. In contrast, *jnk1b* is not required for normal ventricular development and there is no evidence of compensation from *jnk1b* as *MZjnk1a/MZjnk1b* embryos exhibit the same phenotype as *MZjnk1a* embryos.

### Ventricular hypoplasia is due to reduction in FHF ventricular cardiomyocytes

**Fig 6:**
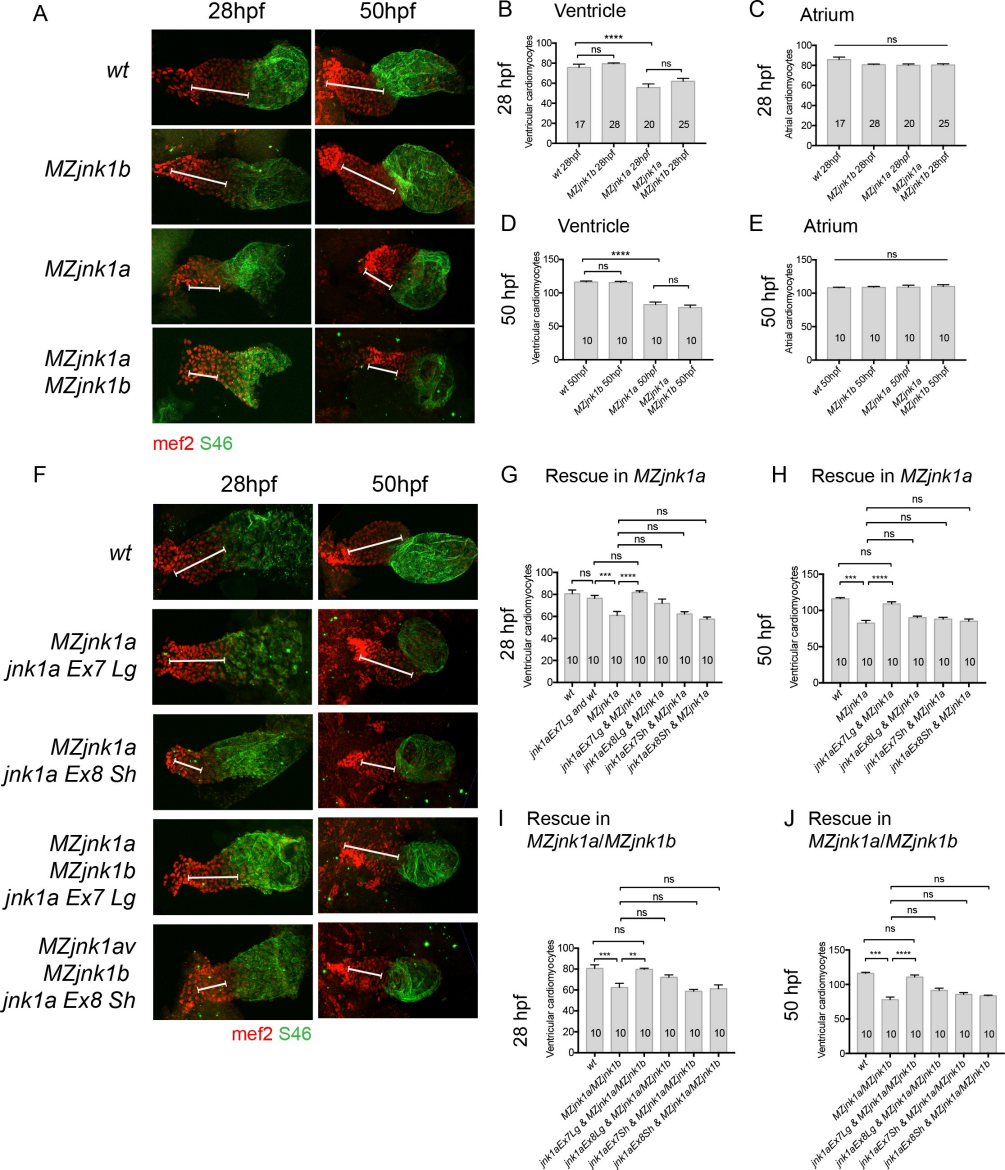
Cardiomyocyte numbers in *MZjnk1a/b* mutants and rescue of deficit with *jnk1a* transcripts. **(A)** Whole mount preparations of hearts from *MZjnk1a/b* mutants at completion of FHF addition (28hpf) and completion of SHF addition (50hpf). All cardiomyocytes are labelled wth mef2 antibody (red) and the atrial cardiomyocytes are labelled with atrial specific S46 antibody (green). Compared to wildtype (wt) embryos there is no reduction in ventricular size in *MZjnk1b* mutants at 28hpf or 50hpf. However the ventricular chamber is reduced in size in *MZjnk1a* null mutants at 28hpf and 50hpf. There is no further change in ventricular chamber size in *MZjnk1a/MZjnk1b* mutants. (**B,D**) Counting of ventricular cardiomyocytes confirms these findings. **(C,E)** There is no alteration in numbers of atrial cardiomyocytes. (**F-J**) Rescue of ventricular phenotype in (G,I) *MZjnk1a* and (H,J) *MZjnk1a/MZjnk1b* embryos with *jnk1a* transcripts at 28hpf (G,H) and 50hpf (I,J). Immunolabelling as in (A). The reduction in ventricular cardiomyocytes in *MZjnk1a* or *MZjnk1a/MZjnk1b* embryos is only reversed by injection of *jnk1a Ex7 Lg* transcript. ** = p<0.02, *** = p<0.001, **** = p<0.0001, ns = not significant.

Following formation of the initial heart tube from FHF cardiomyocytes there is further addition of SHF cells to the ventricle. In septated hearts of mammals and birds these SHF cells form the right ventricle, but in the non-septated zebrafish heart the SHF cells form the distal part of the common ventricle [23,24,33]. In zebrafish it is possible to count the complement of FHF atrial and ventricular cardiomyocytes at 30hpf and the combined FHF and SHF complement at 50hpf (Fig 6A–6E). Examination of *MZjnk1a* embryos revealed a 26% reduction in FHF ventricular cardiomyocytes with normal atrial numbers at 30hpf (Fig 6A–6C). The SHF addition between 30hpf and 50hpf remained normal (Fig 6A, 6D and 6E). There was no cardiomyocyte reduction in *MZjnk1b* mutants and no additional reduction in *MZjnk1a*/*MZjnk1b*, again excluding a role for *jnk1b* in controlling ventricular cardiomyocyte numbers. There was no alteration in atrial cardiomyocyte numbers in these mutants (Fig 6C–6E).

### Only *jnk1a Ex7Lg* transcripts rescue the FHF phenotype

Of the four alternatively spliced *jnk1a* transcripts *Ex7Lg* was most abundant in the heart at 30hpf, but *Ex8Lg*, *Ex7Sh*, and *Ex8Sh* were all present at low levels. We therefore evaluated the potential of all four transcripts to rescue the ventricular phenotype in *MZjnk1a* and *MZjnk1a/MZjnk1b* mutants by injecting 100pg of capped mRNA into one-cell stage embryos (Fig 6F and 6J). The FHF cardiomyocyte deficiency assessed at both 28hpf and 50hpf in *MZjnk1a* mutants (Fig 6G and 6H) was rescued by *jnk1a Ex7Lg*, but not *jnk1a Ex8Lg*, *jnk1a Ex7Sh* and *Jnk1a Ex8Sh*. Identical results were obtained with *MZjnk1a/MZjnk1b* mutants (Fig 6I and 6J). Importantly, injection of 100pg *jnk1a Ex7Lg* into wildtype control embryos had no effect on ventricular cardiomyocyte numbers (Fig 6G).

### The cardiomyocyte deficiency is not due to developmental delay and is already present as cells differentiate

One reason for the reduced ventricular chambers size might be developmental delay. To exclude this, we immunolabelled the forming trunk myotomes with MF20 antibody between 15, 18 and 24 hpf in *MZjnk1a/MZjnk1b* mutants. This demonstrated no difference in the number of myotomes when compared with control embryos (S5 Fig).

**Fig 7:**
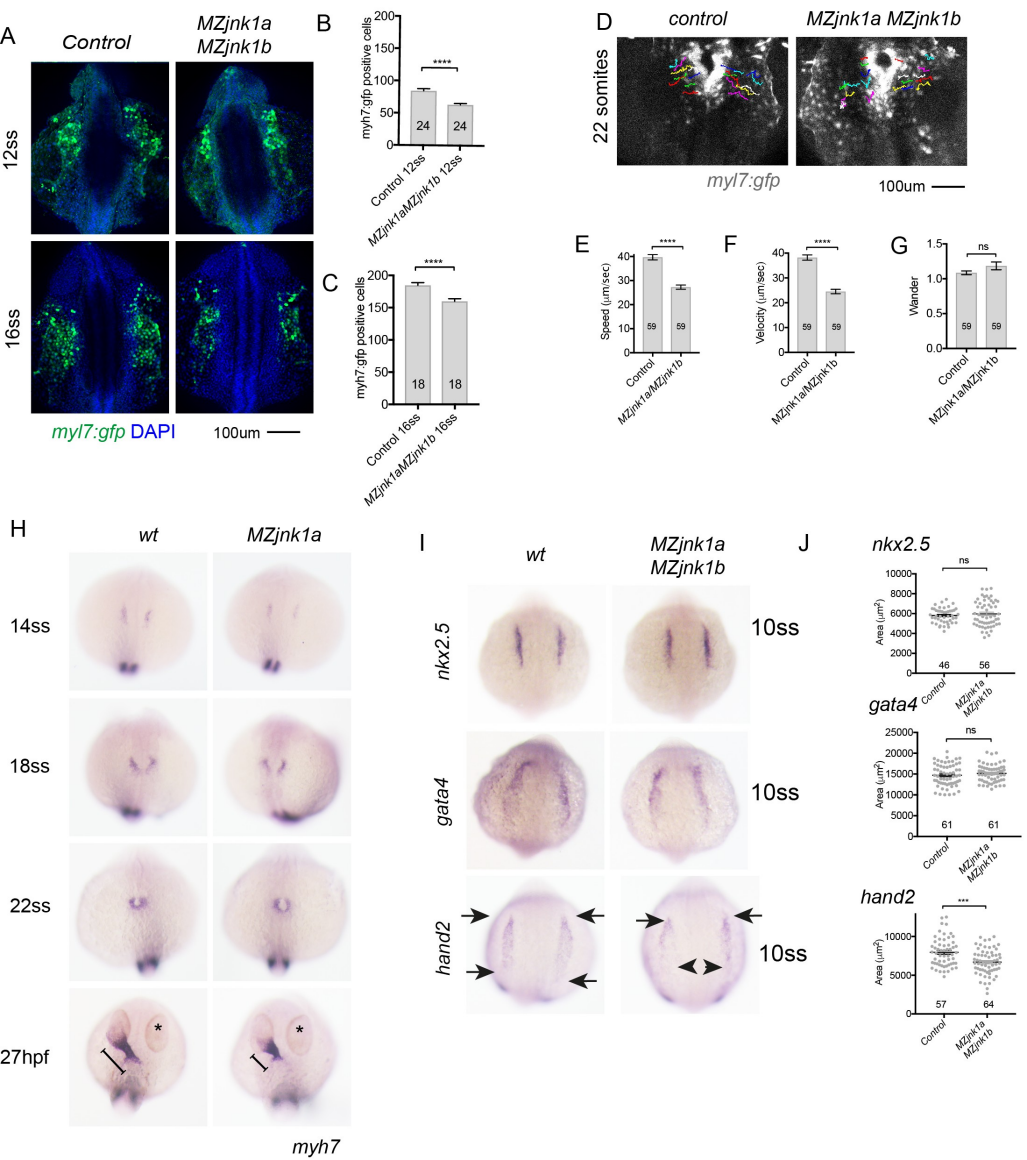
Expression patterns of transcriptional regulators and cardiomyocyte numbers and are disturbed in the ALPM at mid-somitogenesis stages. (**A**) Wholemount preparations of control and *MZjnk1a*/*MZjnk1b* embryos expressing *myl7*:*gfp* transgene. Differentiated cardiomyocytes identified using anti-gfp antibody to detect *myl7;gfp* expression and DAPI to identify nuclei. Reduction in number of cardiomyocyte nuclei at 12ss (**B**) and 16ss (**C**) in *MZjnk1a*/*MZjnk1b* in comparison to control embryos. (**D**) Tracks of cardiomyocytes migrating to form the heart cone in control and *MZjnk1a*/*MZjnk1b* embryos. (**E**) speed, (**F**) velocity and (G) wander index. (**H**) Ventricular cardiomyocytes migrating through the anterior lateral plate mesoderm (ALPM) to form the heart are visualised by WISH for *myh7*. The distribution of cells is identical to the wildtype pattern in *MZjnk1a* null mutants between 14ss and 22ss stages. However at 27hpf the ventricular component of the extended primary heart tube is shorter in *MZjnk1a* null mutants than controls. (**I**) WISH showing expression patterns of *nkx2*.*5*, *gata4* and *hand2* transcription factors in *MZjnk1a*/*MZjnk1b* and control embryos at the 10ss. There are no differences between expression patterns of *nkx2*.*5* or *gata4* between *MZjnk1a*/*MZjnk1b* and control embryos. However, although the anterior exent of *hand2* expression is unchanged, there is a reduction in the posterior expression of *hand2* (arrowheads). (**J**) Area of *nkx2*.*5*, *gata4* and *hand2* gene expression at the 10ss. Only the area of the *hand2* expression is significantly different between *MZjnk1a*/*MZjnk1b* and control embryos. *** = p<0.001. *** = p<0.001, ns = not significant.

We next sought to determine whether normal numbers of cardiomyocytes were being produced within the ALPM. Although ventricular specific *myh7* can only be detected from 14ss, the *myl7*:*gfp* transgene labels both atrial and ventricular cardiomyocytes from the earliest stages of their differentiation when they first appear within the ALPM at the 12ss stage [34]. We therefore took advantage of the *myl7;gfp* transgene available in *MZjnk1a/MZjnk1b* embryos to count cardiomyocyte numbers at 12ss and then when all were differentiated at 16ss (Fig 7A–7C). The cardiomyocyte deficit was already present at 12ss (Fig 7A and 7B) and remained at the same level at 16ss (Fig 7A–7C). Assessment of cell proliferation by BrdU pulsing (S5 Fig) and programmed cell death, by TUNEL (S5 Fig) demonstrated no differences between *MZjnk1a/MZjnk1b* mutants and *wt* controls at 12ss. These data suggest an early role for *jnk1* in FHF ventricular cardiomyocyte production rather than effects on cell proliferation or survival.

### Movement of cardiomyocytes is slowed but cardiomyocytes do not become lost

*Jnk 1* is known to regulate cell migration by phosphorylation of the focal adhesion protein paxillin. Both genetic and pharmacological inhibition of Jnk leads to loss of migration in cultured cells [34] and it is required for Schwann cell migration [35] and in tumour metastasis [36]. We therefore tracked the movement of cardiomyocytes from the ALPM to the primary heart tube [Fig 7D–7G]. In *MZjnk1a/MZjnk1b* mutant embryos cardiomyocytes labelled with the *myl7*:*gfp* transgene were shown to move more slowly than controls [Fig 7E and 7F]. However, the direction of movements was not compromised [Fig 7G]. Despite this slowing of the rate of migration, all cardiomyocytes moved towards the heart and no cells appeared to become isolated from the forming heart [Fig 7D].

Ventricular cardiomyocytes can be first identified by their expression of *myh7*, *the* ventricular cardiomyocyte specific myosin heavy chain transcript from 14ss. To specifically exclude loss of ventricular cardiomyocytes we visualised *myh7* expression by WISH as cardiomyocytes migrated through the anterior lateral plate mesoderm, as they formed the cardiac cone at 18-22ss and at full extension of the primary heart tube at 27hpf. There was no difference in the expression patterns during their migration, and the cardiomyocytes coalesced to form the cardiac cone normally. However, by 27hpf the reduced size of the FHF ventricle was evident (Fig 7H). No ectopic expression, indicating displaced ventricular cardiomyocytes, was observed.

### The expression pattern of *hand2* is reduced in *MZjnk1a*/*MZjnk1b* mutants

Differentiation of FHF cardiomyocytes within the ALPM is dependent on the expression of the key transcriptional regulators *nkx2*.*5*, *hand2* and *gata4*. We examined the expression patterns of these genes by WISH at 10ss and were able to quantify their area of expression in *MZjnk1a/MZjnk1b* mutants and wildtype controls (Fig 7I and 7J). There was no difference in the expression patterns of *nkx2*.*5* and *gata4* between control and *MZjnk1a/MZjnk1b* embryos. However, close examination of *hand2* expression indicated a reduction in the size of the posterior part of the expression field in *MZjnk1a/MZjnk1b* mutants and quantification revealed a significant reduction in the area of *hand2* expression (Fig 7I and 7J).

### *hand2* is sufficient to rescue the cardiomyocyte deficiency in *jnk1a* mutants

**Fig 8:**
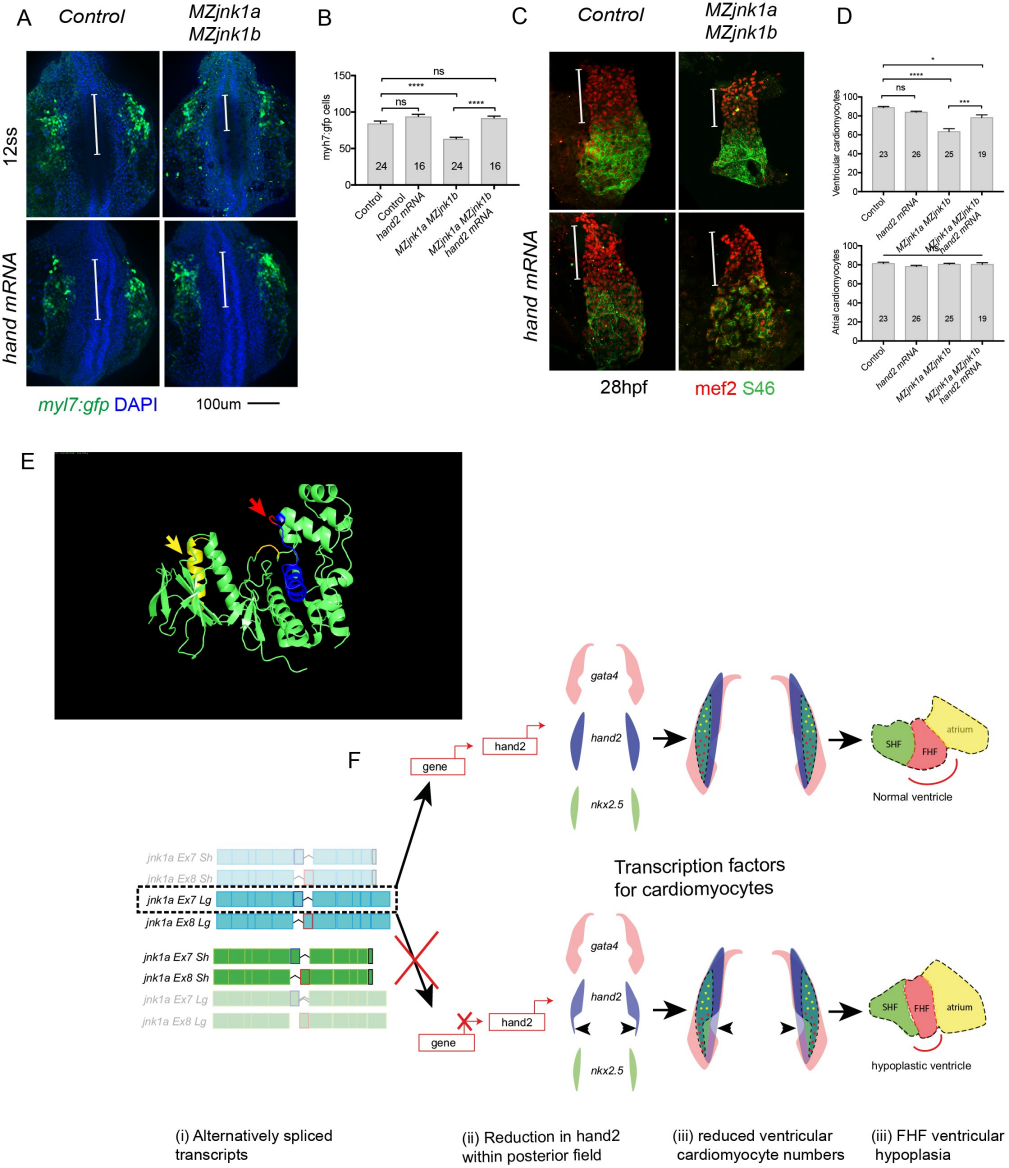
*hand2* mRNA rescues the cardiomyocyte deficiency at 12ss and ventricular cardiomyocyte deficiency at 28hpf. (**A**) Wholemount preparations of control and *MZjnk1a*/*MZjnk1b* embryos labelled with anti-gfp antibody to detect *myl7;gfp*-positive cells and DAPI to identify nuclei at 12ss. The bar indicates the extent of the cardiomyocyte distribution, which is deficient in the posterior part of the field in *MZjnk1a*/*MZjnk1b* embryos but is rescued by *hand2* mRNA. (**B**) Quantification indicates restoration of cardiomyocyte numbers in *MZjnk1a*/*MZjnk1b* embryos injected with *hand2* mRNA. (**C**) At 28 hpf the deficit in FHF ventricular cardiomyocytes (red) in *MZjnk1a*/*MZjnk1b* embryos is rescued with *hand2* mRNA. (**D**) Counting of cardiomyocytes reveals almost complete rescue of ventricular cardiomyocytes with no change in atrial numbers. (**E**) Crystal structure of JNK1 indicating position of C-terminal extension (yellow and yellow arrow) and alternatively spliced exon 6a/b (blue) threonine residue (T228) present in JNK1 Ex6b (red and red arrow). based on crystal structure at https://www.uniprot.org/uniprot/P45983. (**F)** Mechanism of FHF ventricular hypoplasia. (i) Although all 8 possible transcripts are produced by *jnk1a* and j*nk1b*, only *jnk1a Lg* and *jnk1b Sh* forms are expressed at high levels within the heart. (ii) Of these, *jnk1a Ex7Lg* modulates the posterior expression domain of *hand2* (directly or indirectly). (iii) Overlaying the expression fields of transcription factors directly taken from WISH in Fig 7I reveals a reduction in the posterior cardiomyocyte field which gives rise to ventricular cardiomyocytes. (iv) This reduction in cardiomyocytes produces a smaller FHF ventricular segment, with normal SHF ventricular addition which does not compensate for the FHF hypoplasia. * = p<0.05, *** = p<0.001, **** = p<0.0001, ns = not significant.

*hand2* is required sufficient to produce normal ventricular cardiomyocyte numbers [25], but *hand2* overexpression has been shown to abnormally expand the ventricular cardiomyocyte field [26]. Therefore, in attempting to rescue the FHF ventricular hypoplasia in *MZjnk1a/MZjnk1b* null embryos we were careful to use a minimum amount of *hand2* mRNA, that was insufficient to expand ventricular cardiomyocyte numbers in normal embryos (Fig 8A and 8B). When injected into *MZjnk1a/MZjnk1b* null embryos at the 1-cell stage, 80pg of *hand2* mRNA was able to fully restore the number of *myl7*:*gfp* cardiomyocytes at the 12ss (Fig 8A and 8B), and almost completely restore the number of ventricular cardiomyocytes within the heart at 30 hpf (Fig 8C and 8D). Thus, *jnk1a Ex7 Lg* is required to produce the correct numbers of cardiac progenitors that will go on to contribute specifically to the ventricular chamber and this occurs through via regulation of *hand2* expression field.

## Discussion

### *jnk1a* is required for morphogenesis of the FHF ventricular component

Whilst Jnk is acknowledged as a downstream effector of the planar cell polarity pathway, remarkably little is known about the roles it plays in development. The majority of PCP pathway members associated with Jnk are known to be required for heart development [6–10] yet the *Jnk1*,*2*,*3* mouse mutants, singularly or in combination, have not been reported to have disturbances in cardiac development [19,20]. This may be because of redundancy of *Jnk* genes in the mouse or the *Jnk* genes may not be important in heart morphogenesis. Close scrutiny of *Jnk1/Jnk2* null (double mutant) mouse embryos that die at E11.5 with gross apoptosis in the central nervous system, indicates that they had ceased developing at E9.5 and although the embryos appeared normal may have a dilated left (FHF) ventricle [19]. To address the requirement for *Jnk1* in development we turned to zebrafish, although the teleost genome duplication [27] required us to engineer both *jnk1a* and *jnk1b* null mutants. Our studies have shown that development of the FHF proximal ventricular segment in zebrafish is dependent on *jnk1a*. This segment is the developmental equivalent of the mammalian left ventricle which appears abnormal in *Jnk1/Jnk2* null mouse embryos [19]. We suggest that detailed re-analysis of *Jnk1* and *Jnk1/Jnk2* double null mouse embryos is required, ideally utilising Cre-lox to provide knockout solely within the heart progenitor lineages, thus avoiding brain apoptosis [19].

### Alternative splicing and gene expression control *jnk1a/ jnk1b* transcript expression

Through bioinformatic analysis, quantification of transcript expression and developmental studies we have shown there is a specific role for the evolutionarily conserved transcript *jnk1a Ex7Lg*, the equivalent of human *JNK1 Ex6a p54*. In doing so we have shown that the duplication of *JNK1* as *jnk1a* and *jnk1b* in zebrafish has resulted in subspecialisation. Specifically, although both paralogs are widely expressed in the early embryo and heart, only *jnk1a* plays direct roles in cardiac development and *jnk1b* cannot compensate for loss of *jnk1a*. Interestingly, differential function is supported both by changes in the peptide sequence and in overall differential gene expression. For example, *jnk1a* is solely capable of producing transcripts containing the canonical long C-terminal extension during development and *jnk1b* transcripts containing the divergent long C-terminal extension are transcribed only at low levels. The specificity of *jnk1a Ex7Lg* in ventricular cardiac development suggests features within exon 7 and the canonical long C-terminal extension that interact. It is known that Jnk1 becomes activated by phosphorylation of canonical threonine-183/tyrosine-185 residues in the conserved activation loop, which lies in a common part of all jnk1a/jnk1b peptides [Fig 8E]. There are also a series of serine, threonine and tyrosine residues throughout the common peptide sequences, conserved in both jnk1a and jnk1b, that can also be phosphorylated. The functional role of these remains unclear although those with an adjacent proline may be used to phosphorylate other proteins, rather than themselves becoming phosphorylated [37]. Of particular interest is the positioning of the peptides encoded by the alternatively spliced exons 7/8. These sit on the C terminal lobe of the kinase domain and are bound by NOVA2 (a splicing factor), which may direct incorporation of specific exons (discussed in [11]).

Human JNK1 Ex6a and Ex6b are represented by the conserved sequences found in jnk1a Ex7 and jnk1b Ex8 respectively. We know nothing about the JNK1 Ex6a/ jnk1a Ex7 sequence, but the threonine (T228) present in JNK1 Ex6b/jnk1b Ex8 is known to be directly phosphorylated by MAPKK4 during JNK1 activation [37]. It is possible that differential activation by MAPKK4 might be important in distinguishing a stress response from a developmental use of jnk1a. Unfortunately, the crystal structure does not give information on the long C-terminal extension, but it could be brought close to the position of alternatively spiced exons 6a/6b (Ex7/8) on binding to substrate and thus the combination of alternative exon usage with the C-terminal extension may be used in substrate specificity. It is known that the C-terminal extension contains caspase cleavage sites [38] but these are conserved in both the canonical C-terminal extension of jnk1a and the divergent extension of jnk1b.

The jnk1b long C-terminal extension peptide is very different to the jnk1a version, which mirrors that in human JNK1. It is possible that the long C-terminal extension in jnk1b is degenerate because essential functions are still supported by the jnk1a version. Alternatively, it may have taken on an alternative species-specific non-developmental function.

It is known that Jnk1 provides an important stress response to pressure overload in the adult cardiovascular system [39] and both *Jnk1* and *Jnk2* have been implicated in the development of atheromatous plaques [40,41]. The abundance of *jnk1b Ex8Sh* within the heart and the splicing events that favour *jnk1b Ex7Sh* in later heart development suggests that these stress responsive kinases may play roles in the formed heart. It will be interesting to see if the zebrafish regenerative response to injury is dependent on *jnk1b* or other family members, or alternatively if there is upregulation of *jnk1a Ex7Lg* as part of reactivation of the fetal gene program.

### Transcriptional regulation by *Jnk1*

A unique feature of this study has been the functional analysis of individual transcripts by injection into MZ null mutant eggs. This has shown, for the first time, a specific role for the *jnk1a Ex7Lg* alternatively spliced transcript, which is highly conserved in comparison to the human JNK1 Ex6a p54 sequence. Both the Ex7 and the long C-terminal extension are required for ventricular cardiomyocyte specification, indicating a potential three-dimensional interaction between the two domains. Notably, the expression of *jnk1a Ex7Lg* remains higher in the forming heart than in the remainder of the embryo between 28 and 72 hpf. As our studies also show that *jnk1a Ex7Lg* is acting to modulate the *hand2* expression domain prior to the differentiation of FHF ventricular cardiomyocytes, it might be that *jnk1a Ex7Lg* is expressed in progenitor populations rather than differentiated cells. Alternative splicing has already been shown to be involved in maintaining pluripotency and subsequent reprogramming of embryonic stem cells [42] and, of relevance here, the production of cardiac progenitors [43].

Hand (Heart and Neural crest) genes are evolutionarily conserved bHLH factors essential for cardiomyocyte specification [23,44] and in the septated vertebrate heart *Hand1* and *Hand2* genes are found, with mouse *Hand1* providing a similar range of functions to *hand2* in zebrafish, especially relating to FHF cardiomyocyte specification [44]. We have shown that reduced *hand2* expression underlies the reduced specification of FHF ventricular cardiomyocytes in *MZjnk1a/MZjnk1b* mutants and that rescue with small amounts of *hand2* mRNA, insufficient to affect normal wildtype embryos, is able to rescue the *jnk1a* mutant phenotype. This is in keeping with the known permissive role of *hand2* in ventricular cardiomyocyte production [23,24]. Importantly transgenic expression of *Jnk1* mRNA and of a *Jnk1* dominant-negative construct, have shown that *Jnk1* specifically regulates *Hand2* transcription [45]. The stage and mechanism by which *jnk1a* is acting to regulate *hand2* expression remains elusive (Fig 8F). *Jnk* is implicated in a wide range of signalling processes, with several potential partners [11,46] in addition to *c-Jun* [47].

Although it is not clear if direct or indirect regulation of *hand2* activity by *Jnk1a* is taking place, the expression field of *nkx2*.*5*, which contains all FHF cardiomyocytes, remains unchanged in *MZjnk1a* mutants. it seems probable that loss of *jnk1a* restricts the overlapping fields of *hand2* with other transcription factors in the ALPM, reducing the number of FHF progenitors that are capable of differentiation into ventricular cardiomyocytes.

Approaching the question from a different viewpoint, we can ask how might *Jnk* interact with genes already known to bex important in ventricular development? Jnk may activate *AP1* [11] but this is unlikely to be the pathway modulated by *jnk1a Ex7 Lg*, as it has been shown that in zebrafish, *ap1* disturbance appears to affect only the SHF-derived ventricular cardiomyocyte addition [48]. Similarly, fibroblast growth factors [49,50] and retinoic acid (reviewed in [51]) are known to be important in ventricular development, but again seem limited to controlling the SHF additions. It is known that *gata5* is important in specifying ventricular cardiomyocytes in the ALPM [52] and other factors such as *bmp2b* have a more complex regulatory effect, being required for proliferation prior to differentiation, but requiring suppression to allow differentiation [53]. In the future, it will be important to understand the downstream pathway activated by *jnk1a/hand2* during FHF cardiac chamber development.

### Relevance for human and further studies

Population-based genomic studies have been remarkably poor at discovering genetic disturbances that produce non-syndromic congenital heart disease and the favoured explanation is a multigenic aetiology. This study suggests disruption of alternative splicing may be another explanation. Prediction of splicing dysfunction in next generation exome studies is not robust and the reads for Next Generation RNAseq are generally too short to span alterative splicing events. Furthermore, the presence of specific transcripts such as *jnk1a Ex7Lg* may be dependent on splicing factors and sites, for example *Nova2* [11], which are not currently linked with cardiac development.

The highly specific FHF ventricular hypoplasia phenotype observed in zebrafish is the equivalent of left ventricular hypoplasia in the human heart. Untreated, this degree of left ventricular hypoplasia in a septated heart would not be compatible with postnatal life. The reduction in FHF ventricular component is well tolerated in zebrafish because the FHF and SHF addition contribute to the same unseptated chamber. However, this functional tolerance does not result from changes in the SHF ventricular accretion, but instead ongoing growth. Interestingly, there is persisting proximal ventricular dysfunction readily observed at 72 hpf. Although it is difficult to ascribe these functional deficits to specific roles of jnk1a, localised dysfunction may reflect a persisting cell autonomous cardiomyocyte dysfunction due to insufficient *hand2* expression within FHF ventricular myocytes [54]. Specific studies to determine cardiomyocyte size, cell density and single cell transcriptomes will be important to understand this and can be done in this model using transgenes to identify nuclei and cell membranes in cardiomyocytes. The reduction in heart rate may relate to disturbed coupling between the heart field components [26] or may be a manifestation of sympathetic neural dysfunction.

Clinically, left ventricular hypoplasia is often recognised in conjunction with other abnormalities, for example double outlet right ventricle (DORV), atrioventricular septal defect and total anomalous pulmonary venous connections, and often presumed to be a secondary consequence of reduced flow. This *jnk1a* reduction in ventricular size suggests the possibility of a developmental rather than flow-dependent mechanism for left ventricular hypoplasia in these conditions. Left ventricular hypoplasia is a key feature of hypoplastic left heart syndrome (HLHS), where the causes and developmental origins remain obscure [55]. The ability to quantify the FHF and SHF ventricular components in zebrafish, as shown in this manuscript, could provide a functional assay for variants of unknown significance that may cause hypoplasia of the left ventricle. However, it will also be important to characterise the outflow tract phenotype in mice, where hypoplasia of the aorta might support a role in HLHS. Conversely, origin of both great vessels from the right ventricle would support a role in the ventricular hypoplasia seen in double outlet right ventricle [6,56].

Finally, it will also be important to define the pathway of activity for the *jnk1a Ex7Lg* transcript as this is likely to provide new candidate genes that are sufficient to cause left ventricular hypoplasia. These genes may be acting prior to formation of the ventricle and thus may not currently be recognised candidate genes for congenital heart disease.

## Materials and methods

### Animals

Zebrafish were maintained in standard conditions [57] under the Animals (Scientific Procedures) Act 1986, United Kingdom, project license PPL6004548 and conformed to Directive 2010/63/EU of the European Parliament. All experiments were approved by the Newcastle University Animal Welfare and Ethical Review Board. Embryos were obtained from natural pairwise mating, reared in in E3 embryo medium at 28.5°C and staged by somite counting [58]. Some embryos were treated with 1-phenyl 2-thiourea (PTU) from 24 hours post fertilisation (hpf) to suppress pigmentation if needed.

### Mutants

Null mutant *jnk1a* and *jnk1b* zebrafish were generated using CRISPR-Cas9-mediated mutagenesis [59] in the AB wildtype line with guide RNAs identified using Crispr Scan [60]. Screening and subsequent genotyping was carried out by demonstrating disruption of a specific restriction site (*jnk1a*: EcorV and *jnk1b*: MwoI; see S1 Table). Mutations were confirmed by sequencing and outcrossing of lines.

### Cloning of alternatively spliced transcripts

Embryos at 24-72hpf were pooled and RNA extracted using Trizol (Life Technologies) permitting generation of cDNA using Superscript III and oligo-dT primers. Alternatively-spliced transcripts were identified by sub-cloning and sequencing of colonies (see S1 Table for primer sequences).

### Wholemount in-situ hybridisation (WISH)

Full-length *jnk1a* and *jnk1b* alternatively spliced transcripts were used to generate riboprobes. For *jnk2* and *jnk3* an 1100 amplicon was selected. In addition: *hand2*, *nkx2*.*5*, *vmhc* (Deborah Yelon, UCSF, USA), *gata4* (Roger Patient, Oxford University, UK) and *southpaw*, (Steve Wilson, UCL, UK) riboprobes were generously provided by the indicated researchers. Chromogenic WISH was performed according to established protocols [61].

### *Se*mi quantitative RT-PCR splicing assay

Total RNA was extracted from 30–50 pooled embryos or 50 beating hearts [32] and cDNA produced as above. RT-PCR reactions for *jnk1a Ex7* or *Ex8* and *jnk1b Ex7* or *Ex8* were performed in triplicate with Go-taq G2 polymerase (Promega) using conditions: 95°C (2min), [95°C (30sec), 64°C (30sec), 72°C (30sec)] x35 cycles, 72°C (5min). These conditions ensured products obtained were within the linear phase of PCR (S1 Table and S2 Fig). Products were visualised on 2% agarose gel. Aliquots of each PCR product were purified by ethanol and sodium acetate precipitation and resuspended in 10μl of nuclease free water prior to restriction enzyme digestion. Digestion for 1 hour at 37°C was carried out with StyI-HF (R3500, NEB; *jnk1a)* and NheI-HF (R3131, NEB; *jnk1b)* and gel densitometry performed using Fiji [62]. These values were normalized against the *ef1α* value and with regard to the efficiency of the PCR reaction as established with plasmids containing full length transcripts (S2 Fig). Biological statistical replicates (n) were obtained from clutches laid by different pairs of adult fish.

### mRNA injections

All injections were performed at the 1 cell stage. Transcripts for use in rescue experiments were produced using the zebrafish Gateway plasmid system [63]. RNA was produced using the SP6 Ambion mMessage mMachine kit (ThermoFisher Scientific). *hand2* mRNA was generated from plasmid [24] kindly provided by Deborah Yelon, (UCSF). Throughout the *jnk* mRNA rescue experiments a total of 100pg RNA was used, but only 80pg of *hand2* mRNA was injected.

### Immunohistochemistry

Whole-mount immunohistochemistry was performed as previously described [64]. Primary antibodies: chicken anti-GFP (1/500; ab13970, Abcam), mouse anti-MF20 (1/10; MYH1, DSHB), mouse anti-S46 (1/10; MYHCA, DSHB), rabbit anti-Mef2 (1/100; ab646444, Abcam), rat anti-BrdU (1–100; ab6326, Abcam). Secondary antibodies: AlexaFluor-488 anti-mouse (1/300; A-21202, Invitrogen), AlexaFluor-488 anti-mouse IgG1 (1/300; A-21121, Invitrogen), AlexaFluor-488 anti-chicken (1/300; A11039 Invitrogen), AlexaFluor-568 anti-mouse IgG1 (1/300; A-21124, Invitrogen), AlexaFluor-568 anti-mouse IgG2b (1/300; A-21144, Invitrogen), AlexaFluor-568 anti-rabbit (1/300; A10042, Invitrogen), AlexaFluor-568 anti-rat (1/300; A11077, Invitrogen). Nuclei were counterstained with DAPI (1:10,000; D9542, Sigma).

### Cardiomyocyte quantification

Cardiomyocyte nuclei were identified by immunolabelling with anti-Mef2 antibody at 28 and 50hpf [64]. Atrial cardiomyocytes were identified by co-labelling with S46 antibody. Positioning under a glass coverslip allowed all cardiomyocyte nuclei to be imaged in the same focal plane and manual counting was performed using Fiji [62].

### Live imaging of the embryonic heart activity

Embryos were orientated on 1% low melting point agarose containing 0.2% Tricaine. Movies were taken on an inverted microscope with DIC (Nikon Diaphot) using a high-speed digital video camera (640x480 pixels at 127 frames/sec) (Multipix Imaging, Hampshire, UK). Data was analysed using Fiji [62] and simulated M-mode obtained by re-slice through the image series.

### Proliferation assay

Embryos were incubated in 10mM BrdU (B5002, Sigma) in 15% DMSO for 30 min on ice at the 10-somite stage (ss). Then, embryos were washed in E3 medium and allowed to develop until 12ss. After fixing in 4% PFA overnight at 4°C, embryos were stored in methanol at -20°C. Embryos were rehydrated in PBS, then permeabilized using Proteinase K and post-fixed in PFA 4%. After incubation in 2N HCl for 1 hour, standard immunohistochemistry using anti-BrdU and anti-GFP antibodies was carried out. The proportion of BrdU-positive cardiomyocytes was assessed as above.

### Cell death

TUNEL staining (S7165, Merck) was performed as previously described [61] in whole-mount embryos. Counting was carried out using Fiji [62].

### Cardiomyocyte tracking

An established protocol [65] with minor modification was used to track the migration of the cardiomyocytes from the ALPM to the cardiac cone. *myl7*:*gfp* embryos at the 14 ss were hand dechorionated and oriented in 1.5% low melting agarose. A 20-image stack (A1R confocal Microscope, Nikon), using a 20x objective at 3μm intervals, was acquired every 6 minutes between 15–16 ss to 19–20 ss. Manual tracking was performed using Fiji [62] and XY coordinates of individual cardiomyocytes were established for every time point. Based on these coordinates we calculated the following parameters:

Mean speed calculated from each measurable displacement per unit time between the initial (x_i_, y_i_) and the final (x_f_, y_f_) coordinates

$$S=\surd ({\left(x_{2}‑x_{1}\right)}^{2}+{\left(y_{2}‑y_{1}\right)}^{2})+\surd ({\left(x_{3}‑x_{2}\right)}^{2}+{\left(y_{3}‑y_{2}\right)}^{2})+\ldots \surd ({\left(x_{n+1}‑x_{n}\right)}^{2}+{\left(y_{n+1}‑y_{n}\right)}^{2})/total\;time$$

Mean velocity calculated from the absolute displacement per unit time between initial (x_i_, y_i_) and the final (x_f_, y_f_) coordinates

$$V=\surd \left({\left(x_{f}‑x_{i}\right)}^{2}+{\left(y_{f}‑y_{i}\right)}^{2}\right)/total\;time$$

Wandering Index: Ratio between speed and velocity

W=S/V

### Statistics

Sample size was determined with regard to normal values and standard deviations described in previously published experiments. In all cases at least three independent experiments, using embryos from three different clutches of eggs, obtained from different pairs of adult fish were performed. The number of biological replicates, n, is indicated on each graph. Statistics were performed using Graphpad Prism7 for Mac OSX, version 6.0c (Graphpad Software Inc, USA) and, depending on the number of groups to compare, either Student t test or ANOVA with Tukey's post-hoc analysis was performed. Data is presented as mean and SEM.

## Supporting information

S1 Fig Full peptide sequences of duplicated zebrafish *jnk1a* and *jnk1b* inferred from transcripts and compared with human *JNK1*. Black text indicates identical amino acids, green text indicates favourable amino acid substitutions and red text divergent amino acid residues. Exons 2–6 and 9–12 are common to all transcripts and highly conserved. Both *jnk1a* and *jnk1b* genes are capable of producing exon 7 and short C-terminus containing transcripts that fully match human *JNK1* transcripts. However, whilst *Ex8* derived from *jnk1b* matches the human peptide the *Ex8* from *jnk1a* is divergent and contains a serine rather than threonine residue (*). In contrast whilst the long C-terminal extension provided by *jnk1a* matches the human, the *jnk1b* long terminal extension is highly divergent and differs by 9/39 amino acids including insertion of an additional threonine residue.
(TIF)

S2 Fig Validation of splicing assay. (**A**) specificity of primers used in RT-PCR splicing assay. Primers sets to identify *Ex7* and *Ex8* containing variants of both *jnk1a* and *jnk1b* transcripts were assessed against all 8 transcripts contained within plasmids, using primers for the plasmid backbone (M13) as loading control. Restriction enzyme digests showing original PCR product and complete digestion by enzyme to identify C-terminal extension in *jnk1a* and *jnk1b* transcripts. (**B**) Graphical representation of gel densitometry indicating the splicing assay was carried out during the linear phase of amplification for *jnk1a* and *jnk1b Ex7* and *Ex8* PCR (Arbitrary logarithmic Units). (**C**) PCR with plasmid containing transcripts were used as positive controls for PCR reactions and indicated efficiency of each reaction.
(TIF)

S3 Fig WISH for all *jnk1a* and *jnk1b* alternatively spliced transcripts. Chromogenic WISH is not quantitative but indicates the extent of expression. (**A**) Prior to heart formation all transcripts are present throughout the embryo at the start of gastrulation (6hpf) and the end of gastrulation (10hpf). The most abundant transcripts by RT-PCR splicing assay are indicated by dashed lines. (**B**) WISH for all *jnk1a* and *jnk1b* alternatively spliced transcripts at 24hpf and 48hpf. Transcripts with obvious expression within heart—*jnk1a Ex7Lg* and *jnk1b Ex8Sh*—are indicated by arrows. (**C**) Close up of heart in all *jnk1a* transcripts at 24 and 48hpf. Outline of heart indicated by dashed lines. Absent expression indicated by arrow heads and expression in heart indicated by arrows in *jnk1a Ex7Lg*, *jnk1a Ex8Lg* and *jnk1b Ex8Sh*. (**D**) WISH demonstrated expression of *jnk2* and *jnk3* in the developing brain (arrows) but not heart (arrowheads). (**E**) RT-PCR of extracted hearts indicates that *jnk2* is expressed at low level at 24 and 48 hpf, whilst there is late onset of *jnk3* gene expression by 48hpf. *ef1alpha* is used as a loading control.
(TIF)

S4 Fig RT-PCR using primers specific for *jnk1a* and *jnk1b* exon 7 and 8 show nonsense-mediated decay in *MZjnk1a* and *MZjnk1b mutants*, but no up-regulation of other *jnk1* gene products * = p<0.05, ** = p<0.01, *** = p<0.001, **** = p<0.0001. n = 3 clutches in all cases.
(TIF)

S5 Fig Ventricular hypoplasia in *MZjnk1a/MZjnk1b* mutants. (**A**) Cardiac morphology at 28 hpf. All ventricular cardiomyocytes are labelled by MF20 antibody (red) and atrial cells by both MF20 and S46 antibody (appear yellow). Normal appearances seen in wildtype (wt) embryos. Ventricular hypoplasia (indicated by size of white bar) is seen in *MZjnk1a* null mutants but is not seen in *MZjnk1b* null mutants. There is no additional ventricular hypoplasia in *MZjnk1a/MZjnk1b* double mutants. (**B**) MF20 antibody staining to identify somites/skeletal muscle blocks in wild type (wt) and *MZjnk1a/MZjnk1b* mutants (**C**) Somite counting excludes global somatic developmental delay in *MZjnk1a/MZjnk1b* null mutants. (**D,E**) TUNEL labelling at 12ss stage in wild type (wt) and *MZjnk1a/MZjnk1b* mutants. Myocytes in the ALPM are identified by Mef2 antibody. The percentage TUNEL positive cells in Mef2 population is unchanged in *MZjnk1a/MZjnk1b* null mutants. **(F,G)** BrdU incorporation in cardiomyocytes from 10ss to 12ss. Quantification shows that there is no difference in BrdU incorporation index in *MZjnk1a/MZjnk1b* null mutants compared to wild type (wt) controls. ns = nonsignificant.
(TIF)

S1 Table Sequences of oligonucleotides used in this study. Guide RNA sequences of CRISPR Cas9 mutant production and Genotyping primers used for selection of mutants. Transcript cloning indicated are transcript sequences; add additional sequence for Gateway or restriction enzyme cloning. RT-PCR splicing assay primers, see S2 Fig. RT-PCR primers for *jnk2* and *jnk3* and in-situ hybridization primer sequences used to produce sense and non-sense probes.
(DOCX)

S2 Table Nucleotide sequences of alternatively spliced *jnk1a* and *jnk1b* transcripts. Nucleotide sequences for all jnk1a and jnk1b transcripts based on results of bacterial subcloning and translation into peptide sequences.
(DOCX)

S1 Movie Dorsal view of heart in 72hpf *wildtype* control embryo. (MOV)

S2 Movie Dorsal view of heart in 72hpf *MZjnk1a* mutant embryo. (MOV)
