# Supplementary figures and images for: Alternative splicing of jnk1a in zebrafish determines first heart field ventricular cardiomyocyte numbers through modulation of hand2 expression
Source: PLoS Genet. 2020 May 18;16(5):e1008782. doi: 10.1371/journal.pgen.1008782 (PMC7259801; doi:10.1371/journal.pgen.1008782)

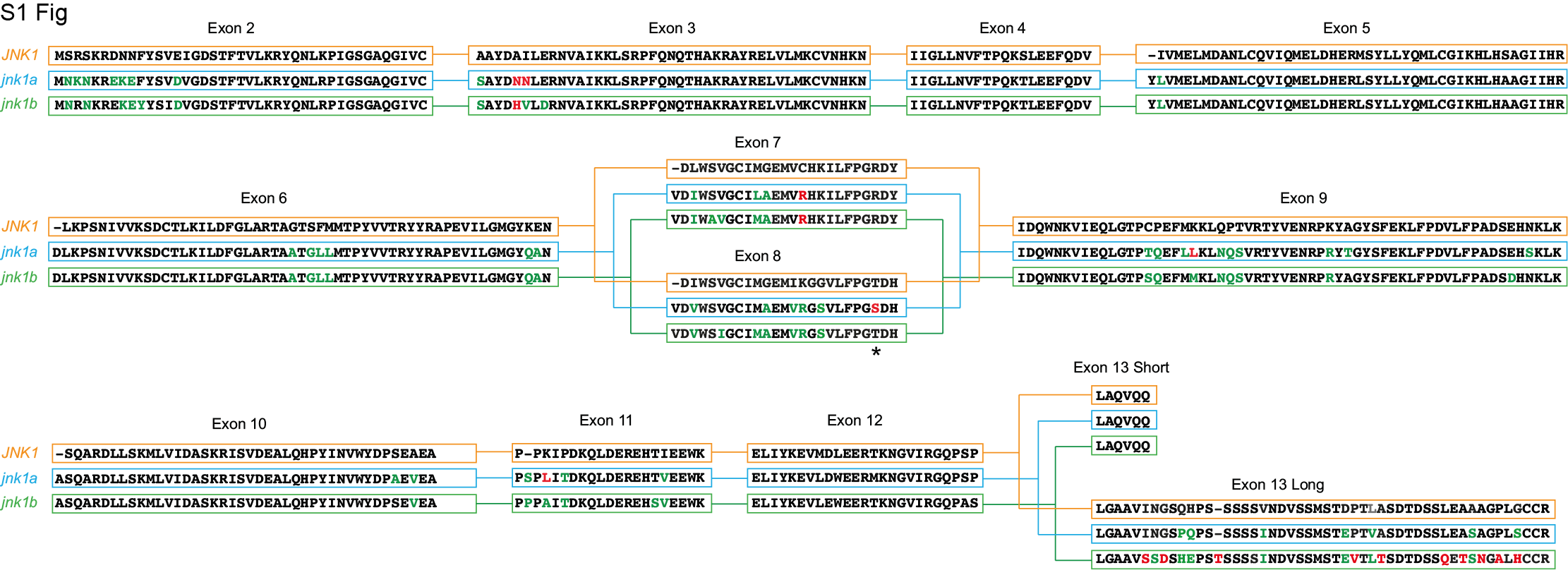

Supplement: S1 Fig — Black text indicates identical amino acids, green text indicates favourable amino acid substitutions and red text divergent amino acid residues. Exons 2–6 and 9–12 are common to all transcripts and highly conserved. Both jnk1a and jnk1b genes are capable of producing exon 7 and short C-terminus containing transcripts that fully match human JNK1 transcripts. However, whilst Ex8 derived from jnk1b matches the human peptide the Ex8 from jnk1a is divergent and contains a serine rather than threonine residue (*). In contrast whilst the long C-terminal extension provided by jnk1a matches the human, the jnk1b long terminal extension is highly divergent and differs by 9/39 amino acids including insertion of an additional threonine residue. (TIF) [file pgen.1008782.s001.tif]

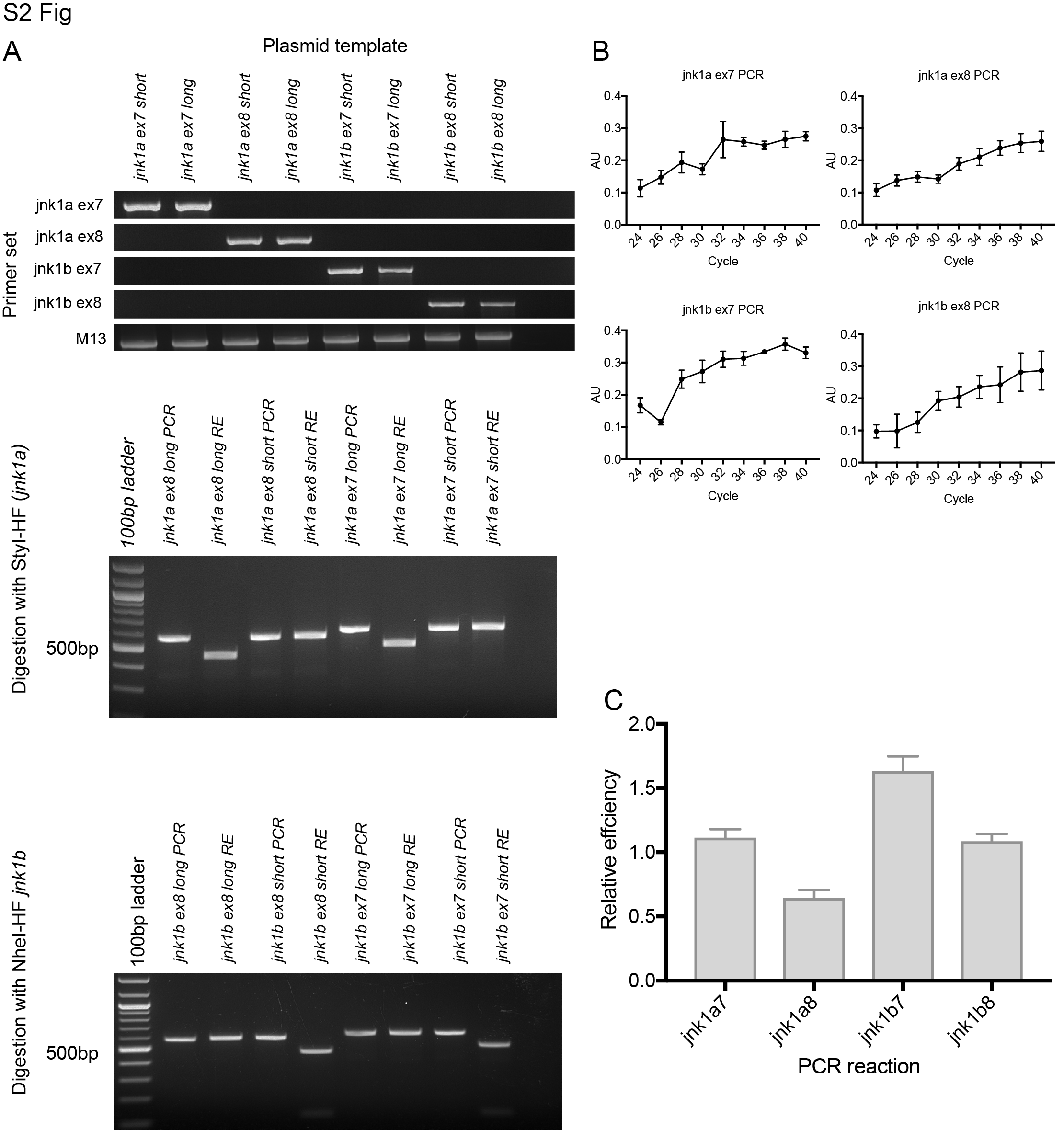

Supplement: S2 Fig — (A) specificity of primers used in RT-PCR splicing assay. Primers sets to identify Ex7 and Ex8 containing variants of both jnk1a and jnk1b transcripts were assessed against all 8 transcripts contained within plasmids, using primers for the plasmid backbone (M13) as loading control. Restriction enzyme digests showing original PCR product and complete digestion by enzyme to identify C-terminal extension in jnk1a and jnk1b transcripts. (B) Graphical representation of gel densitometry indicating the splicing assay was carried out during the linear phase of amplification for jnk1a and jnk1b Ex7 and Ex8 PCR (Arbitrary logarithmic Units). (C) PCR with plasmid containing transcripts were used as positive controls for PCR reactions and indicated efficiency of each reaction. (TIF) [file pgen.1008782.s002.tif]

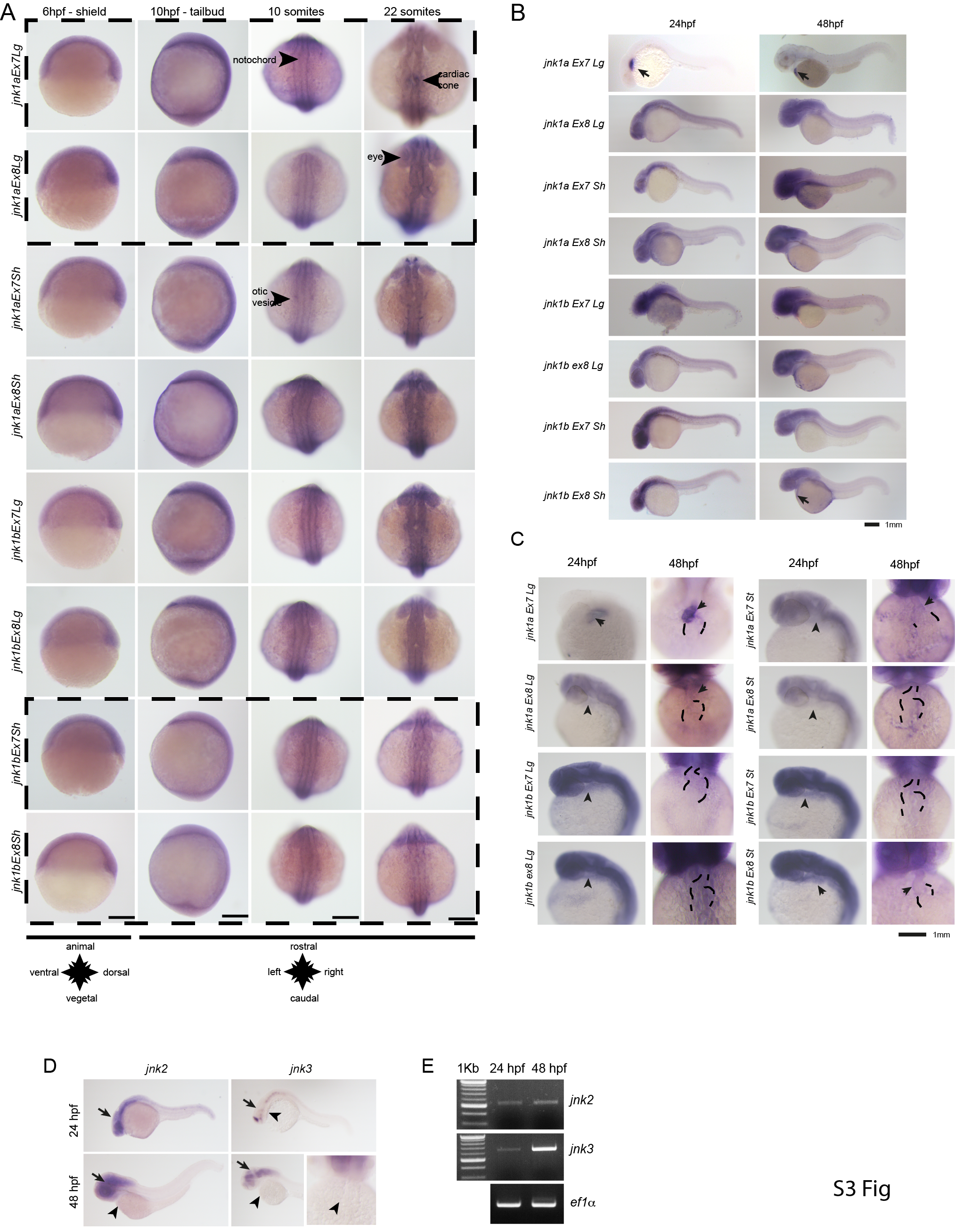

Supplement: S3 Fig — Chromogenic WISH is not quantitative but indicates the extent of expression. (A) Prior to heart formation all transcripts are present throughout the embryo at the start of gastrulation (6hpf) and the end of gastrulation (10hpf). The most abundant transcripts by RT-PCR splicing assay are indicated by dashed lines. (B) WISH for all jnk1a and jnk1b alternatively spliced transcripts at 24hpf and 48hpf. Transcripts with obvious expression within heart—jnk1a Ex7Lg and jnk1b Ex8Sh—are indicated by arrows. (C) Close up of heart in all jnk1a transcripts at 24 and 48hpf. Outline of heart indicated by dashed lines. Absent expression indicated by arrow heads and expression in heart indicated by arrows in jnk1a Ex7Lg, jnk1a Ex8Lg and jnk1b Ex8Sh. (D) WISH demonstrated expression of jnk2 and jnk3 in the developing brain (arrows) but not heart (arrowheads). (E) RT-PCR of extracted hearts indicates that jnk2 is expressed at low level at 24 and 48 hpf, whilst there is late onset of jnk3 gene expression by 48hpf. ef1alpha is used as a loading control. (TIF) [file pgen.1008782.s003.tif]

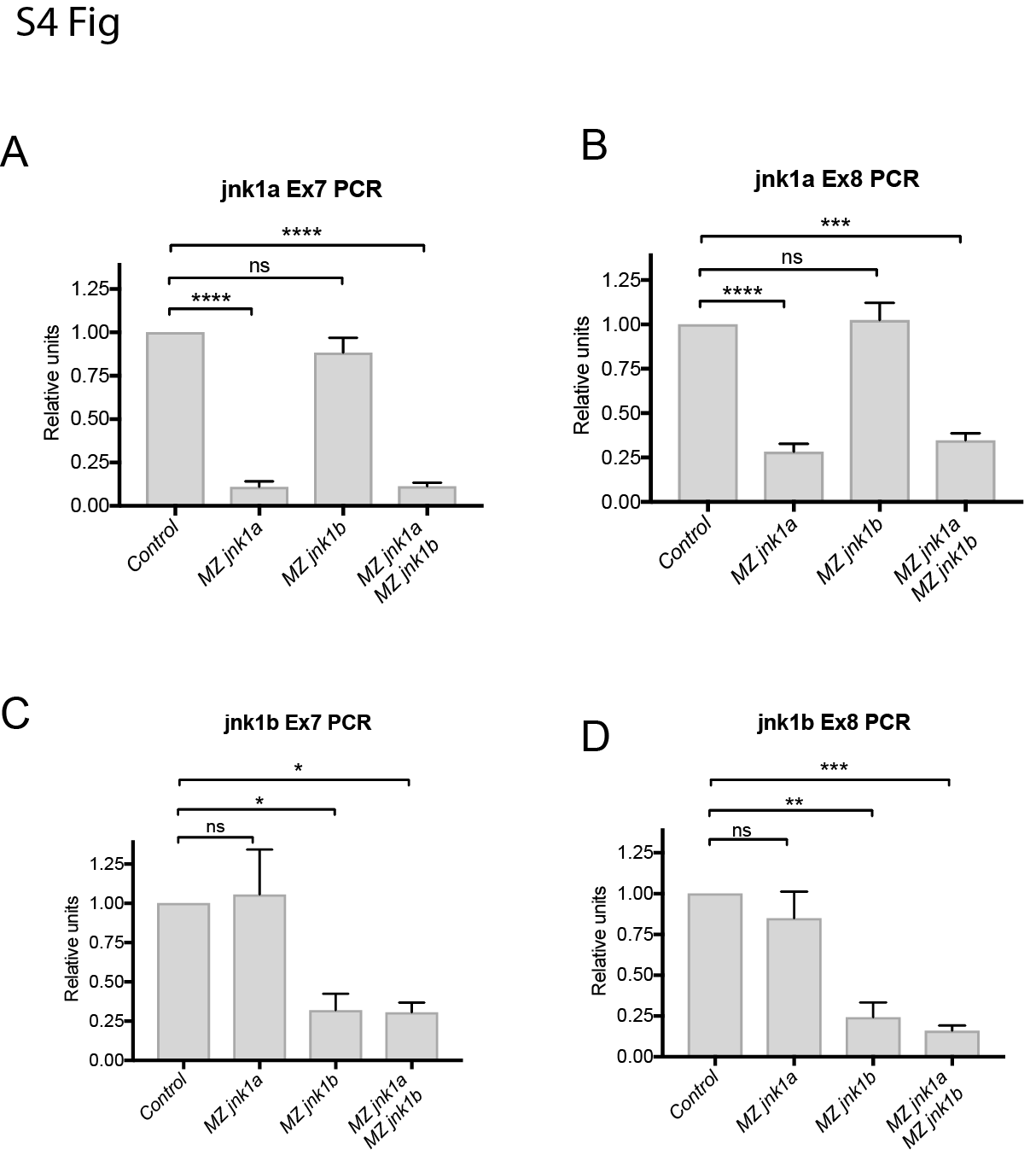

Supplement: S4 Fig — n = 3 clutches in all cases. (TIF) [file pgen.1008782.s004.tif]

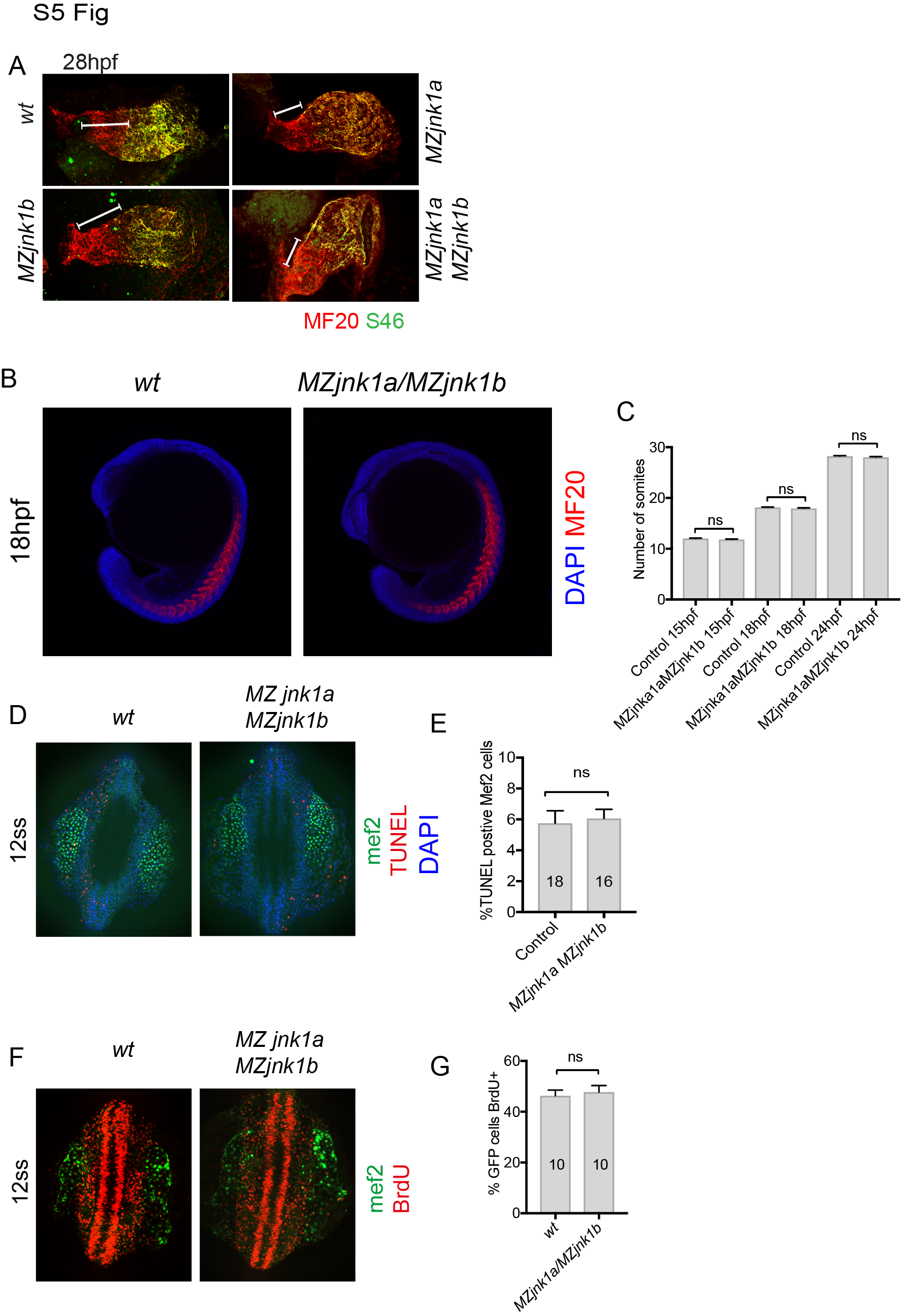

Supplement: S5 Fig — (A) Cardiac morphology at 28 hpf. All ventricular cardiomyocytes are labelled by MF20 antibody (red) and atrial cells by both MF20 and S46 antibody (appear yellow). Normal appearances seen in wildtype (wt) embryos. Ventricular hypoplasia (indicated by size of white bar) is seen in MZjnk1a null mutants but is not seen in MZjnk1b null mutants. There is no additional ventricular hypoplasia in MZjnk1a/MZjnk1b double mutants. (B) MF20 antibody staining to identify somites/skeletal muscle blocks in wild type (wt) and MZjnk1a/MZjnk1b mutants (C) Somite counting excludes global somatic developmental delay in MZjnk1a/MZjnk1b null mutants. (D,E) TUNEL labelling at 12ss stage in wild type (wt) and MZjnk1a/MZjnk1b mutants. Myocytes in the ALPM are identified by Mef2 antibody. The percentage TUNEL positive cells in Mef2 population is unchanged in MZjnk1a/MZjnk1b null mutants. (F,G) BrdU incorporation in cardiomyocytes from 10ss to 12ss. Quantification shows that there is no difference in BrdU incorporation index in MZjnk1a/MZjnk1b null mutants compared to wild type (wt) controls. ns = nonsignificant. (TIF) [file pgen.1008782.s005.tif]
