# Supplementary material for: Alternative splicing of jnk1a in zebrafish determines first heart field ventricular cardiomyocyte numbers through modulation of hand2 expression
Source: PLoS Genet. 2020 May 18;16(5):e1008782. doi: 10.1371/journal.pgen.1008782 (PMC7259801; doi:10.1371/journal.pgen.1008782)
Supplement: S1 Table — Guide RNA sequences of CRISPR Cas9 mutant production and Genotyping primers used for selection of mutants. Transcript cloning indicated are transcript sequences; add additional sequence for Gateway or restriction enzyme cloning. RT-PCR splicing assay primers, see S2 Fig. RT-PCR primers for jnk2 and jnk3 and in-situ hybridization primer sequences used to produce sense and non-sense probes. (DOCX) [file pgen.1008782.s006.docx]

**Supplementary table1. Sequences of oligonucleotides used in study**

| **GuideRNA** | |
| --- | --- |
| *jnk1a gRNA* | ATTTAGGTGACACTATAGGAATGGGATATCAAGCCAAGTTTTAGAGCTAGAAATAGCAAG |
| *jnk1b gRNA* | ATTTAGGTGACACTATAGGAGGCCGGTTGCAGCCGTTGTTTTAGAGCTAGAAATAGCAAG |

| **Genotyping primers** | |
| --- | --- |
| *jnk1a^n1^ fwd* | GCTACAGGTCTGCTGATGACAC |
| *jnk1a^n1^ rev* | TTTTGATGCTGAAACCACAAAG |
| *jnk1b^n2^ fwd* | TTTTCCTAGGATCTGAAACCA |
| *jnk1b^n2^ rev* | CCGTTTGGTGAGATCTGTTTTC |

| **Transcript cloning** | |
| --- | --- |
| *jnk1a ATG fwd* | ATGAACAAAAATAAGCGAGA |
| *jnk1a STOP long rev* | TCATCTGCAGCAGCTCAGGG |
| *jnk1b ATG fwd* | ATGAACAGGAATAAGCGCGA |
| *jnk1b STOP long rev* | TCATCTGCAGCAGTGCAGCG |
| *jnk1a and b STOP short common rev* | TCACTGCTGCACCTGTGCTA |

|  | **RT-PCR splicing assay** |
| --- | --- |
| *jnk1a ex7 fwd* | TGTGGACATTTGGTCTGTGG |
| *jnk1a ex8 fwd* | TTTCCGGGTTCAGATCATATTGA |
| *jnk1a common rev* | AAGCTGCTGTCTGTGTCTGA |
| *jnk1b ex7 fwd* | ACGTGGATATTTGGGCTGTTG |
| *jnk1b ex8 fwd* | AGTGTGTTGTTTCCTGGCAC |
| *jnk1b common rev* | ACTGCTGTCGGTGTCTGAG |
| *ef1αforw* | CTTCTCAGGCTGACTGTGC |
| *ef1αrev* | CCGCTAGCATTACCCTCC |

|  | **RT-PCR** |
| --- | --- |
| *jnk2 fwd* | CCACCTTTACCGTCCTCAGA |
| *jnk2 rev* | CAATGCAAGAGCTGTCGGTA |
| *jnk3 fwd* | ACCTTCACGGTTCTCAAACG |
| *jnk3 rev* | CCCTTCACCACACCATTCTT |

|  | ***In situ* hybridization** |
| --- | --- |
| *jnk2 fwd-T7* | taatacgactcactatagggCTCCACCTTTACCGTCCTCA |
| *jnk2 rev-T3* | attaaccctcactaaagggaCGTCGACATGGATGAGATGT |
| *jnk3 fwd-T7* | taatacgactcactatagggTGAGCAAAAGCAAAGTGGAC |
| *jnk3 rev-T3* | attaaccctcactaaagggaCTCTCCTCGAAGTTCATCACC |
