# Supplementary material for: Alternative splicing of jnk1a in zebrafish determines first heart field ventricular cardiomyocyte numbers through modulation of hand2 expression
Source: PLoS Genet. 2020 May 18;16(5):e1008782. doi: 10.1371/journal.pgen.1008782 (PMC7259801; doi:10.1371/journal.pgen.1008782)
Supplement: S2 Table — Nucleotide sequences for all jnk1a and jnk1b transcripts based on results of bacterial subcloning and translation into peptide sequences. (DOCX) [file pgen.1008782.s007.docx]

**Supplementary table 2**

**Sequences of jnk1a and jnk1b alternatively spliced transcripts.**

>jnk1a_ex7_sh

ATGAACAAAAATAAGCGAGAGAAAGAATTCTACAGTGTAGATGTGGGGGATTCGACATTCACAGTGTTGAAGCGCTATCAGAATCTAAGACCCATTGGCTCGGGTGCTCAGGGAATAGTCTGCTCAGCGTATGACAACAACCTCGAGCGAAACGTGGCTATAAAGAAACTCAGCCGGCCTTTTCAAAATCAAACTCATGCCAAACGTGCGTACAGAGAGCTGGTGCTCATGAAATGTGTCAACCATAAAAATATAATAGGCCTCTTAAATGTTTTTACACCGCAAAAAACATTAGAAGAATTCCAAGATGTTTACCTAGTAATGGAGCTGATGGATGCGAACCTCTGCCAAGTCATTCAGATGGAGCTGGACCACGAGCGGCTGTCCTATCTGCTGTACCAGATGCTGTGTGGAATAAAACACCTCCACGCGGCGGGGATCATCCACAGGGACCTGAAACCCAGTAACATCGTGGTAAAGTCAGACTGTACCCTGAAGATCCTGGATTTCGGTCTGGCGCGGACAGCAGCTACAGGTCTGCTGATGACACCATATGTGGTGACCCGTTACTACAGAGCCCCTGAAGTCATCCTGGGAATGGGATATCAAGCCAATGTGGACATTTGGTCTGTGGGCTGCATTTTGGCAGAAATGGTCCGTCACAAAATCCTTTTTCCTGGGAGGGACTATATTGATCAGTGGAATAAAGTAATAGAGCAGCTGGGAACGCCAACTCAGGAGTTCTTGTTGAAACTCAACCAGTCTGTGCGGACCTATGTGGAGAACAGGCCCCGGTACACTGGATATAGCTTTGAGAAGCTGTTTCCTGATGTCCTGTTCCCTGCTGATTCAGAACACAGCAAACTAAAAGCGAGTCAGGCGCGGGACCTGCTGTCTAAAATGCTGGTGATTGATGCATCAAAACGAATCTCGGTGGATGAGGCTTTGCAGCACCCCTACATTAACGTGTGGTACGACCCGGCTGAAGTGGAAGCGCCTTCTCCTCTGATCACAGACAAACAGCTCGATGAGAGGGAACACACAGTGGAAGAGTGGAAAGAACTGATCTATAAAGAAGTGCTGGATTGGGAAGAACGGATGAAGAACGGTGTTATTCGAGGTCAGCCCTCCCCCCTAGCACAGGTGCAGCAGTGA

> jnk1a_ex7_sh Protein

MNKNKREKEFYSVDVGDSTFTVLKRYQNLRPIGSGAQGIVCSAYDNNLERNVAIKKLSRPFQNQTHAKRAYRELVLMKCVNHKNIIGLLNVFTPQKTLEEFQDVYLVMELMDANLCQVIQMELDHERLSYLLYQMLCGIKHLHAAGIIHRDLKPSNIVVKSDCTLKILDFGLARTAATGLLMTPYVVTRYYRAPEVILGMGYQANVDIWSVGCILAEMVRHKILFPGRDYIDQWNKVIEQLGTPTQEFLLKLNQSVRTYVENRPRYTGYSFEKLFPDVLFPADSEHSKLKASQARDLLSKMLVIDASKRISVDEALQHPYINVWYDPAEVEAPSPLITDKQLDEREHTVEEWKELIYKEVLDWEERMKNGVIRGQPSPLAQVQQ*

>jnk1a_ex7_Lg

ATGAACAAAAATAAGCGAGAGAAAGAATTCTACAGTGTAGATGTGGGGGATTCGACATTCACAGTGTTGAAGCGCTATCAGAATCTAAGACCCATTGGCTCGGGTGCTCAGGGAATAGTCTGCTCAGCGTATGACAACAACCTCGAGCGAAACGTGGCTATAAAGAAACTCAGCCGGCCTTTTCAAAATCAAACTCATGCCAAACGTGCGTACAGAGAGCTGGTGCTCATGAAATGTGTCAACCATAAAAATATAATAGGCCTCTTAAATGTTTTTACACCGCAAAAAACATTAGAAGAATTCCAAGATGTTTACCTAGTAATGGAGCTGATGGATGCGAACCTCTGCCAAGTCATTCAGATGGAGCTGGACCACGAGCGGCTGTCCTATCTGCTGTACCAGATGCTGTGTGGAATAAAACACCTCCACGCGGCGGGGATCATCCACAGGGACCTGAAACCCAGTAACATCGTGGTAAAGTCAGACTGTACCCTGAAGATCCTGGATTTCGGTCTGGCGCGGACAGCAGCTACAGGTCTGCTGATGACACCATATGTGGTGACCCGTTACTACAGAGCCCCTGAAGTCATCCTGGGAATGGGATATCAAGCCAATGTGGACATTTGGTCTGTGGGCTGCATTTTGGCAGAAATGGTCCGTCACAAAATCCTTTTTCCTGGGAGGGACTATATTGATCAGTGGAATAAAGTAATAGAGCAGCTGGGAACGCCAACTCAGGAGTTCTTGTTGAAACTCAACCAGTCTGTGCGGACCTATGTGGAGAACAGGCCCCGGTACACTGGATATAGCTTTGAGAAGCTGTTTCCTGATGTCCTGTTCCCTGCTGATTCAGAACACAGCAAACTAAAAGCGAGTCAGGCGCGGGACCTGCTGTCTAAAATGCTGGTGATTGATGCATCAAAACGAATCTCGGTGGATGAGGCTTTGCAGCACCCCTACATTAACGTGTGGTACGACCCGGCTGAAGTGGAAGCGCCTTCTCCTCTGATCACAGACAAACAGCTCGATGAGAGGGAACACACAGTGGAAGAGTGGAAAGAACTGATCTATAAAGAAGTGCTGGATTGGGAAGAACGGATGAAGAACGGTGTTATTCGAGGTCAGCCCTCCCCCCTAGGTGCAGCAGTGATCAACGGCTCACCCCAGCCCTCATCCTCATCCTCCATCAACGACGTGTCCTCCATGTCCACAGAGCCCACCGTGGCCTCAGACACAGACAGCAGCTTAGAGGCCTCGGCGGGACCCCTGAGCTGCTGCAGATGA

> jnk1a_ex7_Lg Protein

MNKNKREKEFYSVDVGDSTFTVLKRYQNLRPIGSGAQGIVCSAYDNNLERNVAIKKLSRPFQNQTHAKRAYRELVLMKCVNHKNIIGLLNVFTPQKTLEEFQDVYLVMELMDANLCQVIQMELDHERLSYLLYQMLCGIKHLHAAGIIHRDLKPSNIVVKSDCTLKILDFGLARTAATGLLMTPYVVTRYYRAPEVILGMGYQANVDIWSVGCILAEMVRHKILFPGRDYIDQWNKVIEQLGTPTQEFLLKLNQSVRTYVENRPRYTGYSFEKLFPDVLFPADSEHSKLKASQARDLLSKMLVIDASKRISVDEALQHPYINVWYDPAEVEAPSPLITDKQLDEREHTVEEWKELIYKEVLDWEERMKNGVIRGQPSPLGAAVINGSPQPSSSSSINDVSSMSTEPTVASDTDSSLEASAGPLSCCR*

>jnk1a_ex8_sh

ATGAACAAAAATAAGCGAGAGAAAGAATTCTACAGTGTAGATGTGGGGGATTCGACATTCACAGTGTTGAAGCGCTATCAGAATCTAAGACCCATTGGCTCGGGTGCTCAGGGAATAGTCTGCTCAGCGTATGACAACAACCTCGAGCGAAACGTGGCTATAAAGAAACTCAGCCGGCCTTTTCAAAATCAAACTCATGCCAAACGTGCGTACAGAGAGCTGGTGCTCATGAAATGTGTCAACCATAAAAATATAATAGGCCTCTTAAATGTTTTTACACCGCAAAAAACATTAGAAGAATTCCAAGATGTTTACCTAGTAATGGAGCTGATGGATGCGAACCTCTGCCAAGTCATTCAGATGGAGCTGGACCACGAGCGGCTGTCCTATCTGCTGTACCAGATGCTGTGTGGAATAAAACACCTCCACGCGGCGGGGATCATCCACAGGGACCTGAAACCCAGTAACATCGTGGTAAAGTCAGACTGTACCCTGAAGATCCTGGATTTCGGTCTGGCGCGGACAGCAGCTACAGGTCTGCTGATGACACCATATGTGGTGACCCGTTACTACAGAGCCCCTGAAGTCATCCTGGGAATGGGATATCAAGCCAATGTGGATGTGTGGTCTGTCGGCTGTATCATGGCTGAAATGGTCAGAGGTAGTGTATTATTTCCGGGTTCAGATCATATTGATCAGTGGAATAAAGTAATAGAGCAGCTGGGAACGCCAACTCAGGAGTTCTTGTTGAAACTCAACCAGTCTGTGCGGACCTATGTGGAGAACAGGCCCCGGTACACTGGATATAGCTTTGAGAAGCTGTTTCCTGATGTCCTGTTCCCTGCTGATTCAGAACACAGCAAACTAAAAGCGAGTCAGGCGCGGGACCTGCTGTCTAAAATGCTGGTGATTGATGCATCAAAACGAATCTCGGTGGATGAGGCTTTGCAGCACCCCTACATTAACGTGTGGTACGACCCGGCTGAAGTGGAAGCGCCTTCTCCTCTGATCACAGACAAACAGCTCGATGAGAGGGAACACACAGTGGAAGAGTGGAAAGAACTGATCTATAAAGAAGTGCTGGATTGGGAAGAACGGATGAAGAACGGTGTTATTCGAGGTCAGCCCTCCCCCCTAGCACAGGTGCAGCAGTGA

> jnk1a_ex8_sh protein

MNKNKREKEFYSVDVGDSTFTVLKRYQNLRPIGSGAQGIVCSAYDNNLERNVAIKKLSRPFQNQTHAKRAYRELVLMKCVNHKNIIGLLNVFTPQKTLEEFQDVYLVMELMDANLCQVIQMELDHERLSYLLYQMLCGIKHLHAAGIIHRDLKPSNIVVKSDCTLKILDFGLARTAATGLLMTPYVVTRYYRAPEVILGMGYQANVDVWSVGCIMAEMVRGSVLFPGSDHIDQWNKVIEQLGTPTQEFLLKLNQSVRTYVENRPRYTGYSFEKLFPDVLFPADSEHSKLKASQARDLLSKMLVIDASKRISVDEALQHPYINVWYDPAEVEAPSPLITDKQLDEREHTVEEWKELIYKEVLDWEE

>jnk1a_ex8_Lg

ATGAACAAAAATAAGCGAGAGAAAGAATTCTACAGTGTAGATGTGGGGGATTCGACATTCACAGTGTTGAAGCGCTATCAGAATCTAAGACCCATTGGCTCGGGTGCTCAGGGAATAGTCTGCTCAGCGTATGACAACAACCTCGAGCGAAACGTGGCTATAAAGAAACTCAGCCGGCCTTTTCAAAATCAAACTCATGCCAAACGTGCGTACAGAGAGCTGGTGCTCATGAAATGTGTCAACCATAAAAATATAATAGGCCTCTTAAATGTTTTTACACCGCAAAAAACATTAGAAGAATTCCAAGATGTTTACCTAGTAATGGAGCTGATGGATGCGAACCTCTGCCAAGTCATTCAGATGGAGCTGGACCACGAGCGGCTGTCCTATCTGCTGTACCAGATGCTGTGTGGAATAAAACACCTCCACGCGGCGGGGATCATCCACAGGGACCTGAAACCCAGTAACATCGTGGTAAAGTCAGACTGTACCCTGAAGATCCTGGATTTCGGTCTGGCGCGGACAGCAGCTACAGGTCTGCTGATGACACCATATGTGGTGACCCGTTACTACAGAGCCCCTGAAGTCATCCTGGGAATGGGATATCAAGCCAATGTGGATGTGTGGTCTGTCGGCTGTATCATGGCTGAAATGGTCAGAGGTAGTGTATTATTTCCGGGTTCAGATCATATTGATCAGTGGAATAAAGTAATAGAGCAGCTGGGAACGCCAACTCAGGAGTTCTTGTTGAAACTCAACCAGTCTGTGCGGACCTATGTGGAGAACAGGCCCCGGTACACTGGATATAGCTTTGAGAAGCTGTTTCCTGATGTCCTGTTCCCTGCTGATTCAGAACACAGCAAACTAAAAGCGAGTCAGGCGCGGGACCTGCTGTCTAAAATGCTGGTGATTGATGCATCAAAACGAATCTCGGTGGATGAGGCTTTGCAGCACCCCTACATTAACGTGTGGTACGACCCGGCTGAAGTGGAAGCGCCTTCTCCTCTGATCACAGACAAACAGCTCGATGAGAGGGAACACACAGTGGAAGAGTGGAAAGAACTGATCTATAAAGAAGTGCTGGATTGGGAAGAACGGATGAAGAACGGTGTTATTCGAGGTCAGCCCTCCCCCCTAGGTGCAGCAGTGATCAACGGCTCACCCCAGCCCTCATCCTCATCCTCCATCAACGACGTGTCCTCCATGTCCACAGAGCCCACCGTGGCCTCAGACACAGACAGCAGCTTAGAGGCCTCGGCGGGACCCCTGAGCTGCTGCAGATGA

> jnk1a_ex8_Lg Protein

MNKNKREKEFYSVDVGDSTFTVLKRYQNLRPIGSGAQGIVCSAYDNNLERNVAIKKLSRPFQNQTHAKRAYRELVLMKCVNHKNIIGLLNVFTPQKTLEEFQDVYLVMELMDANLCQVIQMELDHERLSYLLYQMLCGIKHLHAAGIIHRDLKPSNIVVKSDCTLKILDFGLARTAATGLLMTPYVVTRYYRAPEVILGMGYQANVDVWSVGCIMAEMVRGSVLFPGSDHIDQWNKVIEQLGTPTQEFLLKLNQSVRTYVENRPRYTGYSFEKLFPDVLFPADSEHSKLKASQARDLLSKMLVIDASKRISVDEALQHPYINVWYDPAEVEAPSPLITDKQLDEREHTVEEWKELIYKEVLDWEERMKNGVIRGQPSPLGAAVINGSPQPSSSSSINDVSSMSTEPTVASDTDSSLEASAGPLSCCR*

>jnk1b_ex7_sh

ATGAACAGGAATAAGCGCGAGAAAGAATATTACAGCATAGATGTAGGAGATTCGACGTTCACCGTTTTGAAGCGCTATCAGAATTTAAGACCAATCGGGTCCGGAGCACAAGGCATCGTCTGCTCAGCGTATGACCACGTCCTCGATCGAAATGTGGCGATTAAGAAACTCAGCCGACCCTTTCAAAACCAAACTCATGCCAAACGGGCCTACAGAGAACTGGTCCTGATGAAATGCGTCAACCACAAAAATATAATTGGCTTACTAAACGTGTTCACACCACAGAAGACCCTTGAAGAGTTCCAGGATGTTTATCTGGTGATGGAGCTGATGGATGCAAACCTGTGTCAGGTGATTCAGATGGAGCTGGACCACGAGAGGCTGTCCTACCTGCTCTATCAGATGCTCTGCGGCATTAAACACCTGCACGCTGCTGGCATCATACACAGGGACCTGAAACCCAGTAATATAGTAGTGAAATCGGACTGCACGCTGAAGATCCTGGATTTCGGTCTGGCCAGAACGGCTGCAACCGGCCTCCTCATGACTCCTTATGTAGTGACACGCTATTATCGGGCCCCAGAGGTCATCCTGGGCATGGGTTATCAAGCTAACGTGGATATTTGGGCTGTTGGCTGCATTATGGCAGAGATGGTGCGGCACAAAATCCTTTTTCCAGGGAGGGACTATATTGACCAGTGGAATAAAGTGATCGAGCAGCTCGGCACGCCGTCACAGGAGTTCATGATGAAGCTGAATCAGTCTGTGAGGACGTATGTGGAGAACCGGCCTCGGTATGCGGGATACAGCTTTGAGAAGCTCTTCCCAGACGTGCTCTTCCCCGCAGACTCGGACCACAACAAACTCAAGGCGAGTCAGGCACGAGACTTGTTATCCAAAATGCTGGTAATAGATGCGTCCAAGCGGATCTCTGTAGACGAGGCGCTTCAGCACCCCTACATCAACGTTTGGTACGACCCGTCAGAAGTGGAGGCGCCACCACCAGCGATCACGGATAAACAGCTCGATGAGAGAGAACACTCAGTGGAAGAGTGGAAAGAGCTCATATATAAGGAAGTGCTGGAATGGGAGGAGCGAACAAAAAATGGAGTGATCAGAGGACAGCCGGCCTCGCTAGCACAGGTGCAGCAGTGA

> jnk1b_ex7_sh Protein

MNRNKREKEYYSIDVGDSTFTVLKRYQNLRPIGSGAQGIVCSAYDHVLDRNVAIKKLSRPFQNQTHAKRAYRELVLMKCVNHKNIIGLLNVFTPQKTLEEFQDVYLVMELMDANLCQVIQMELDHERLSYLLYQMLCGIKHLHAAGIIHRDLKPSNIVVKSDCTLKILDFGLARTAATGLLMTPYVVTRYYRAPEVILGMGYQANVDIWAVGCIMAEMVRHKILFPGRDYIDQWNKVIEQLGTPSQEFMMKLNQSVRTYVENRPRYAGYSFEKLFPDVLFPADSDHNKLKASQARDLLSKMLVIDASKRISVDEALQHPYINVWYDPSEVEAPPPAITDKQLDEREHSVEEWKELIYKEVLEWEERTKNGVIRGQPASLAQVQQ*

>jnk1b_ex7_Lg

ATGAACAGGAATAAGCGCGAGAAAGAATATTACAGCATAGATGTAGGAGATTCGACGTTCACCGTTTTGAAGCGCTATCAGAATTTAAGACCAATCGGGTCCGGAGCACAAGGCATCGTCTGCTCAGCGTATGACCACGTCCTCGATCGAAATGTGGCGATTAAGAAACTCAGCCGACCCTTTCAAAACCAAACTCATGCCAAACGGGCCTACAGAGAACTGGTCCTGATGAAATGCGTCAACCACAAAAATATAATTGGCTTACTAAACGTGTTCACACCACAGAAGACCCTTGAAGAGTTCCAGGATGTTTATCTGGTGATGGAGCTGATGGATGCAAACCTGTGTCAGGTGATTCAGATGGAGCTGGACCACGAGAGGCTGTCCTACCTGCTCTATCAGATGCTCTGCGGCATTAAACACCTGCACGCTGCTGGCATCATACACAGGGACCTGAAACCCAGTAATATAGTAGTGAAATCGGACTGCACGCTGAAGATCCTGGATTTCGGTCTGGCCAGAACGGCTGCAACCGGCCTCCTCATGACTCCTTATGTAGTGACACGCTATTATCGGGCCCCAGAGGTCATCCTGGGCATGGGTTATCAAGCTAACGTGGATATTTGGGCTGTTGGCTGCATTATGGCAGAGATGGTGCGGCACAAAATCCTTTTTCCAGGGAGGGACTATATTGACCAGTGGAATAAAGTGATCGAGCAGCTCGGCACGCCGTCACAGGAGTTCATGATGAAGCTGAATCAGTCTGTGAGGACGTATGTGGAGAACCGGCCTCGGTATGCGGGATACAGCTTTGAGAAGCTCTTCCCAGACGTGCTCTTCCCCGCAGACTCGGACCACAACAAACTCAAGGCGAGTCAGGCACGAGACTTGTTATCCAAAATGCTGGTAATAGATGCGTCCAAGCGGATCTCTGTAGACGAGGCGCTTCAGCACCCCTACATCAACGTTTGGTACGACCCGTCAGAAGTGGAGGCGCCACCACCAGCGATCACGGATAAACAGCTCGATGAGAGAGAACACTCAGTGGAAGAGTGGAAAGAGCTCATATATAAGGAAGTGCTGGAATGGGAGGAGCGAACAAAAAATGGAGTGATCAGAGGACAGCCGGCCTCGCTAGGTGCAGCAGTGAGCAGTGACTCCCATGAGCCCTCGACGTCGTCCTCCTCCATAAACGATGTGTCGTCCATGTCCACCGAGGTCACGCTGACCTCAGACACCGACAGCAGTCAGGAGACGTCCAACGGAGCGCTGCACTGCTGCAGATGA

> jnk1b_ex7_Lg Protein

MNRNKREKEYYSIDVGDSTFTVLKRYQNLRPIGSGAQGIVCSAYDHVLDRNVAIKKLSRPFQNQTHAKRAYRELVLMKCVNHKNIIGLLNVFTPQKTLEEFQDVYLVMELMDANLCQVIQMELDHERLSYLLYQMLCGIKHLHAAGIIHRDLKPSNIVVKSDCTLKILDFGLARTAATGLLMTPYVVTRYYRAPEVILGMGYQANVDIWAVGCIMAEMVRHKILFPGRDYIDQWNKVIEQLGTPSQEFMMKLNQSVRTYVENRPRYAGYSFEKLFPDVLFPADSDHNKLKASQARDLLSKMLVIDASKRISVDEALQHPYINVWYDPSEVEAPPPAITDKQLDEREHSVEEWKELIYKEVLEWEERTKNGVIRGQPASLGAAVSSDSHEPSTSSSSINDVSSMSTEVTLTSDTDSSQETSNGALHCCR*

>jnk1b_ex8_sh

ATGAACAGGAATAAGCGCGAGAAAGAATATTACAGCATAGATGTAGGAGATTCGACGTTCACCGTTTTGAAGCGCTATCAGAATTTAAGACCAATCGGGTCCGGAGCACAAGGCATCGTCTGCTCAGCGTATGACCACGTCCTCGATCGAAATGTGGCGATTAAGAAACTCAGCCGACCCTTTCAAAACCAAACTCATGCCAAACGGGCCTACAGAGAACTGGTCCTGATGAAATGCGTCAACCACAAAAATATAATTGGCTTACTAAACGTGTTCACACCACAGAAGACCCTTGAAGAGTTCCAGGATGTTTATCTGGTGATGGAGCTGATGGATGCAAACCTGTGTCAGGTGATTCAGATGGAGCTGGACCACGAGAGGCTGTCCTACCTGCTCTATCAGATGCTCTGCGGCATTAAACACCTGCACGCTGCTGGCATCATACACAGGGACCTGAAACCCAGTAATATAGTAGTGAAATCGGACTGCACGCTGAAGATCCTGGATTTCGGTCTGGCCAGAACGGCTGCAACCGGCCTCCTCATGACTCCTTATGTAGTGACACGCTATTATCGGGCCCCAGAGGTCATCCTGGGCATGGGTTATCAAGCTAACGTTGATGTCTGGTCTATTGGCTGCATCATGGCTGAAATGGTCAGAGGTAGTGTGTTGTTTCCTGGCACAGACCATATTGACCAGTGGAATAAAGTGATCGAGCAGCTCGGCACGCCGTCACAGGAGTTCATGATGAAGCTGAATCAGTCTGTGAGGACGTATGTGGAGAACCGGCCTCGGTATGCGGGATACAGCTTTGAGAAGCTCTTCCCAGACGTGCTCTTCCCCGCAGACTCGGACCACAACAAACTCAAGGCGAGTCAGGCACGAGACTTGTTATCCAAAATGCTGGTAATAGATGCGTCCAAGCGGATCTCTGTAGACGAGGCGCTTCAGCACCCCTACATCAACGTTTGGTACGACCCGTCAGAAGTGGAGGCGCCACCACCAGCGATCACGGATAAACAGCTCGATGAGAGAGAACACTCAGTGGAAGAGTGGAAAGAGCTCATATATAAGGAAGTGCTGGAATGGGAGGAGCGAACAAAAAATGGAGTGATCAGAGGACAGCCGGCCTCGCTAGCACAGGTGCAGCAGTGA

> jnk1b_ex8_sh Protein

MNRNKREKEYYSIDVGDSTFTVLKRYQNLRPIGSGAQGIVCSAYDHVLDRNVAIKKLSRPFQNQTHAKRAYRELVLMKCVNHKNIIGLLNVFTPQKTLEEFQDVYLVMELMDANLCQVIQMELDHERLSYLLYQMLCGIKHLHAAGIIHRDLKPSNIVVKSDCTLKILDFGLARTAATGLLMTPYVVTRYYRAPEVILGMGYQANVDVWSIGCIMAEMVRGSVLFPGTDHIDQWNKVIEQLGTPSQEFMMKLNQSVRTYVENRPRYAGYSFEKLFPDVLFPADSDHNKLKASQARDLLSKMLVIDASKRISVDEALQHPYINVWYDPSEVEAPPPAITDKQLDEREHSVEEWKELIYKEVLEWEERTKNGVIRGQPASLAQVQQ*

>jnk1b_ex8_Lg

ATGAACAGGAATAAGCGCGAGAAAGAATATTACAGCATAGATGTAGGAGATTCGACGTTCACCGTTTTGAAGCGCTATCAGAATTTAAGACCAATCGGGTCCGGAGCACAAGGCATCGTCTGCTCAGCGTATGACCACGTCCTCGATCGAAATGTGGCGATTAAGAAACTCAGCCGACCCTTTCAAAACCAAACTCATGCCAAACGGGCCTACAGAGAACTGGTCCTGATGAAATGCGTCAACCACAAAAATATAATTGGCTTACTAAACGTGTTCACACCACAGAAGACCCTTGAAGAGTTCCAGGATGTTTATCTGGTGATGGAGCTGATGGATGCAAACCTGTGTCAGGTGATTCAGATGGAGCTGGACCACGAGAGGCTGTCCTACCTGCTCTATCAGATGCTCTGCGGCATTAAACACCTGCACGCTGCTGGCATCATACACAGGGACCTGAAACCCAGTAATATAGTAGTGAAATCGGACTGCACGCTGAAGATCCTGGATTTCGGTCTGGCCAGAACGGCTGCAACCGGCCTCCTCATGACTCCTTATGTAGTGACACGCTATTATCGGGCCCCAGAGGTCATCCTGGGCATGGGTTATCAAGCTAACGTTGATGTCTGGTCTATTGGCTGCATCATGGCTGAAATGGTCAGAGGTAGTGTGTTGTTTCCTGGCACAGACCATATTGACCAGTGGAATAAAGTGATCGAGCAGCTCGGCACGCCGTCACAGGAGTTCATGATGAAGCTGAATCAGTCTGTGAGGACGTATGTGGAGAACCGGCCTCGGTATGCGGGATACAGCTTTGAGAAGCTCTTCCCAGACGTGCTCTTCCCCGCAGACTCGGACCACAACAAACTCAAGGCGAGTCAGGCACGAGACTTGTTATCCAAAATGCTGGTAATAGATGCGTCCAAGCGGATCTCTGTAGACGAGGCGCTTCAGCACCCCTACATCAACGTTTGGTACGACCCGTCAGAAGTGGAGGCGCCACCACCAGCGATCACGGATAAACAGCTCGATGAGAGAGAACACTCAGTGGAAGAGTGGAAAGAGCTCATATATAAGGAAGTGCTGGAATGGGAGGAGCGAACAAAAAATGGAGTGATCAGAGGACAGCCGGCCTCGCTAGGTGCAGCAGTGAGCAGTGACTCCCATGAGCCCTCGACGTCGTCCTCCTCCATAAACGATGTGTCGTCCATGTCCACCGAGGTCACGCTGACCTCAGACACCGACAGCAGTCAGGAGACGTCCAACGGAGCGCTGCACTGCTGCAGATGA

> jnk1b_ex8_Lg Protein

MNRNKREKEYYSIDVGDSTFTVLKRYQNLRPIGSGAQGIVCSAYDHVLDRNVAIKKLSRPFQNQTHAKRAYRELVLMKCVNHKNIIGLLNVFTPQKTLEEFQDVYLVMELMDANLCQVIQMELDHERLSYLLYQMLCGIKHLHAAGIIHRDLKPSNIVVKSDCTLKILDFGLARTAATGLLMTPYVVTRYYRAPEVILGMGYQANVDVWSIGCIMAEMVRGSVLFPGTDHIDQWNKVIEQLGTPSQEFMMKLNQSVRTYVENRPRYAGYSFEKLFPDVLFPADSDHNKLKASQARDLLSKMLVIDASKRISVDEALQHPYINVWYDPSEVEAPPPAITDKQLDEREHSVEEWKELIYKEVLEWEERTKNGVIRGQPASLGAAVSSDSHEPSTSSSSINDVSSMSTEVTLTSDTDSSQETSNGALHCCR*
